# Systematic engineering of synthetic minimal promoters for tunable transgene expression in mammalian cells

**DOI:** 10.1093/nar/gkag1052

**Published:** 2026-11-04

**Authors:** Masha Zaitsev-Chaikovsky, Shira Shalev, Tali Lanton, Yarden Dalyahu, Lior Nissim

**Affiliations:** Department of Biochemistry and Molecular Biology, Institute for Medical Research Israel-Canada, Faculty of Medicine, The Hebrew University of Jerusalem, Jerusalem 91120, Israel; Department of Biochemistry and Molecular Biology, Institute for Medical Research Israel-Canada, Faculty of Medicine, The Hebrew University of Jerusalem, Jerusalem 91120, Israel; Department of Biochemistry and Molecular Biology, Institute for Medical Research Israel-Canada, Faculty of Medicine, The Hebrew University of Jerusalem, Jerusalem 91120, Israel; Department of Biochemistry and Molecular Biology, Institute for Medical Research Israel-Canada, Faculty of Medicine, The Hebrew University of Jerusalem, Jerusalem 91120, Israel; Department of Biochemistry and Molecular Biology, Institute for Medical Research Israel-Canada, Faculty of Medicine, The Hebrew University of Jerusalem, Jerusalem 91120, Israel

## Abstract

Fine-tuning transgene levels is crucial in fundamental and applied research. Native promoters are broadly utilized to regulate transgene expression. However, their large size and structural complexity hinder predictable tuning, constrain compatibility with viral vectors with limited capacity, and increase the risk of homologous recombination. Synthetic promoters are short *de novo* sequences, typically consisting of an enhancer region, which is positioned upstream of a minimal promoter that determines transcription initiation efficiency. Enhancer optimization typically yields coarse activation shifts, alters response curves, and requires case-by-case optimization. Synthetic minimal promoter architecture enables fine-tuning that is potentially compatible with any enhancer region. In this study, we systematically optimized minimal promoter sequences, core element composition, and regulatory motif sequences. Our findings support a modular model of minimal promoter function in which *cis-*regulatory elements from different minimal promoters can be mixed, altered, added, or deleted to yield predictable expression outcomes. We shortened and thereby enhanced the efficiency of known minimal promoters, engineered modular tunable minimal promoters, and generated candidate regulatory elements spanning 7–8 nucleotides that drive efficient transcription when combined with artificial ${5^{\prime}}$ untranslated regions (5'UTRs) that function as an additional regulatory layer. This framework broadens the utility of synthetic promoters for next-generation applications.

## Graphical Abstract

**Graphical Abstract:**
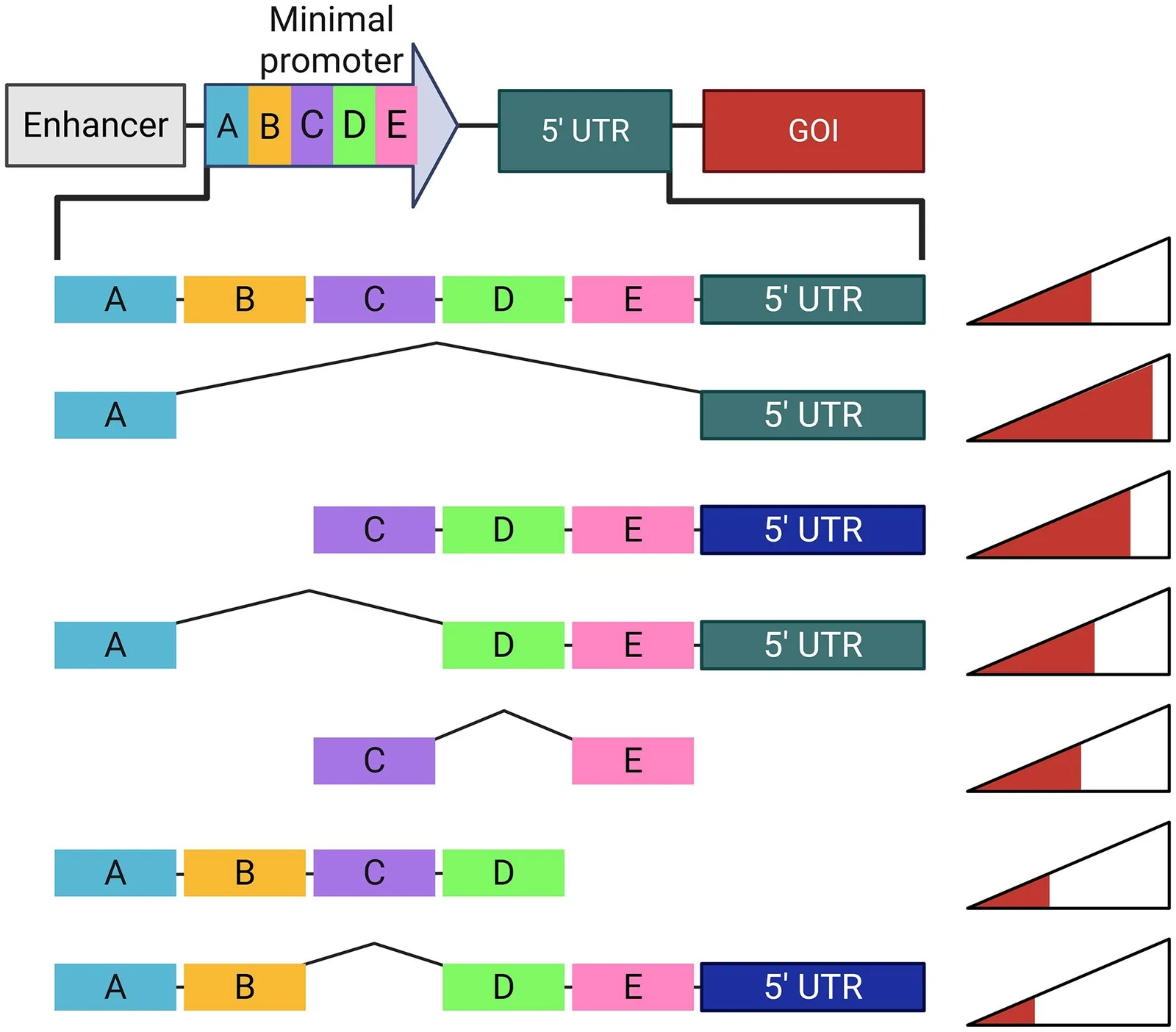

## Introduction

Gene expression typically spans broad dynamic ranges and is characterized by non-linear responses to protein concentrations. Therefore, fine-tuning transgene expression is critical for fundamental and applied research, including studies of regulatory dynamics, metabolic engineering, and gene therapies [1–5]. For example, transgene expression must be sufficient to achieve therapeutic efficacy while avoiding overexpression-mediated toxicity, a requirement that is particularly critical in applications with narrow therapeutic windows, such as gene therapies [6, 7]. Post-transcriptional and post-translational strategies enable tunable protein levels, including AU-rich motifs or ${3^{\prime}}$ untranslated regions (${3^{\prime}}$ UTRs) that regulate mRNA stability [8, 9], synthetic intronic microRNA loops for dosage control [10], engineered ribosome efficiency [11, 12], and degron libraries that modulate protein half-life [13]. However, post-transcriptional and post-translational strategies operate downstream of transcription and do not provide direct control over gene expression. Therefore, promoter-based regulation remains an essential mechanism to directly and predictably govern the magnitude, timing, and cell-type specificity of transgene expression [14, 15].

Native promoters are broadly utilized for transgene regulation, including cell-state-specific promoters that restrict gene expression to specific cellular conditions [16], viral promoters that drive potent expression across most cell types but are prone to silencing [17, 18], and constitutive mammalian promoters that support moderate but persistent expression [19–21]. However, the application and tunability of such promoters are limited due to their size and structural complexity [22, 23]. Native promoters typically span hundreds to thousands of base pairs, thereby reducing the encoding space available for transgenes in viral vectors with limited encoding capacity [24–27] and increasing the risk of homologous recombination with host genomes [5]. Furthermore, mammalian promoters lack clearly defined boundaries and often depend on distal regulatory elements, leading to context-dependent variability in transgene expression when encoded outside their genomic location [28–30]. Finally, the heterogeneous *cis-*regulatory composition of native promoters often leads to leaky expression that impairs cell-type specificity [31–33]. Consequently, predictable tuning of native promoter activity remains challenging and requires optimization for each promoter individually. These limitations underscore the need for a generalized strategy to tune the activity of compact promoters, which are suited for precise transgene regulation, reduce recombination risk, and improve compatibility with size-restricted viral vectors [25, 26, 34].

Synthetic biology is focused on the engineering of artificial biological building blocks, which can be assembled into gene circuits with novel functions [35–40]. Synthetic promoters are *de novo* sequences that commonly span 100–250 base pairs and are engineered for diverse functionalities, including inducible [41–46], strong, and cell-state-specific expression [33, 46]. Their design typically includes an enhancer region comprised of *cis-*regulatory elements that recruit transcription factors and co-activators, positioned upstream of a minimal promoter that provides the core motifs required for transcription initiation [46–49] (Fig. 1A). Therefore, both the enhancer region and minimal promoter regions can be modulated to tune synthetic promoter activity [50–55]. The enhancer region can be modulated by varying the sequence, number, or arrangement of transcription-factor binding sites [46, 48, 56–59]. For example, massively parallel reporter assays have enabled programmable promoter functionalities in mammalian cells, including high inducibility and low basal activity [52, 60]. Similar strategies have been applied in other organisms. In yeast, upstream activating sequences function as enhancer-like regulatory elements, and their transcription-factor binding sites have been added, removed, or rearranged to program condition-specific expression [61]. In bacteria, operator sites for ligand-responsive transcription factors have been combined with core promoter elements to tune promoter strength and dynamic range [62]. However, enhancer-based strategies typically yield coarse shifts in activation, alter response curves, and require case-by-case optimization.

**Figure 1.**
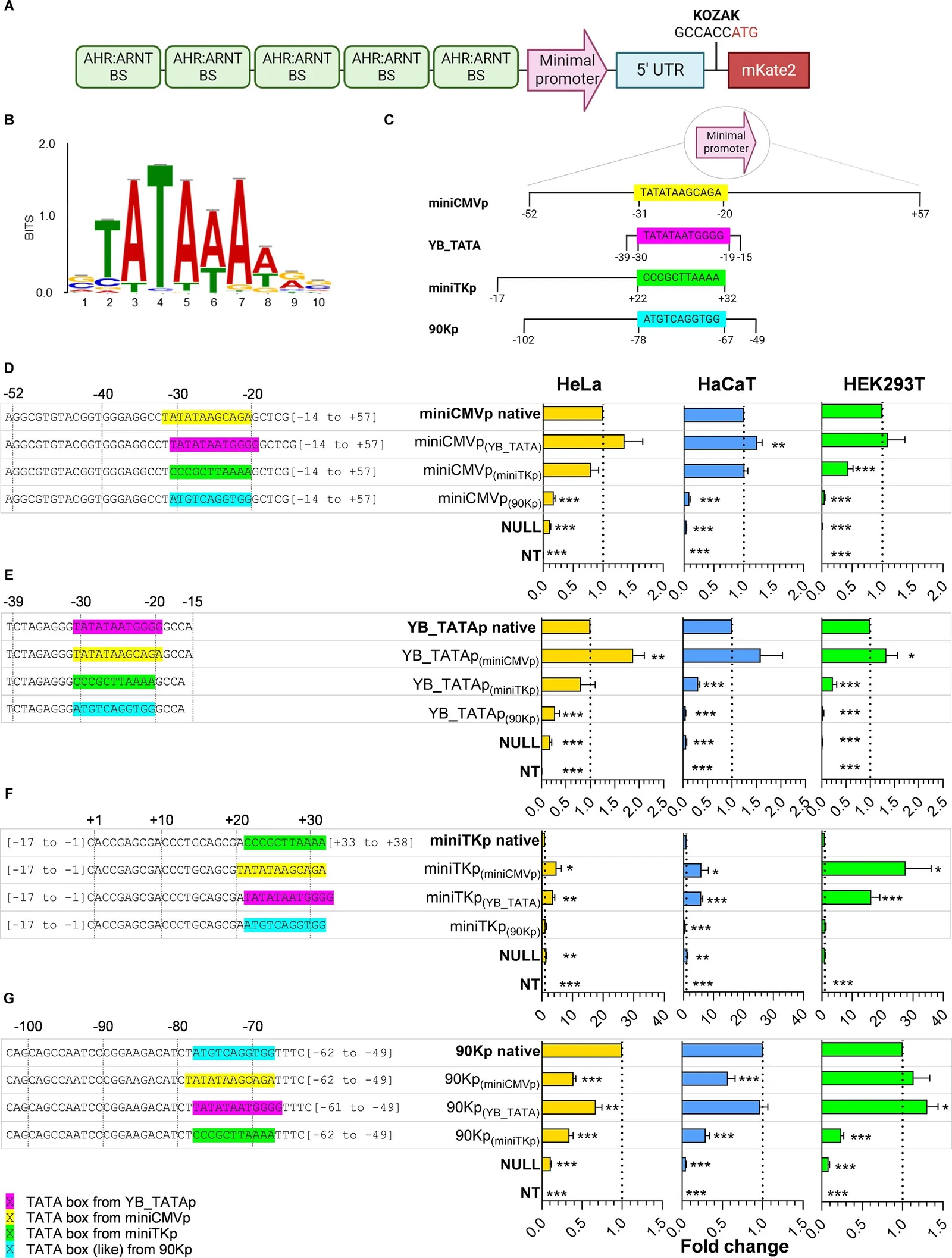
TATA box swapping between minimal promoters reveals context-dependent effects on promoter activity. (**A**) Synthetic promoter design used for all experiments. Synthetic enhancer and mKate2 reporter backbone applied to all constructs. The synthetic enhancer region contains five binding sites of the AHR:ARNT transcription factor complex (GGGGATCGCGTGACAACCC) positioned in tandem with 3 bp spacers (full sequence in Table 2). Downstream is the RND ${5^{\prime}}$UTR (Table 6), a KOZAK sequence, and the mKate2 reporter gene. (**B**) TATA box consensus across mammalian promoters [80]. (**C**) Schematic overview illustrating the architecture of four minimal promoters employed in the TATA box swapping analysis (Table 1), depicted according to their relative positions within each promoter. (**D**) miniCMVp variants. (**E**) YB_TATAp variants. (**F**) miniTKp variants. (**G**) 90Kp variants across HeLa, HaCaT, and HEK293T cells. All minimal promoter variants are annotated relative to the native minimal promoter’s transcription start site (TSS; +1). All four panels display the weighted median of mKate2 expressed under each native or chimeric minimal promoter construct, normalized to each minimal promoter's native construct (Table 1); full promoter variations sequences in Table 3. NULL denotes the lack of a minimal promoter within the synthetic enhancer–reporter construct; NT denotes non-treated, naïve cells; and square brackets indicate additional downstream nucleotides in the minimal promoter, with the position of non-displayed nucleotides shown in brackets (full sequences in Tables 1 and 3). Data are presented as the mean ± SEM; *n* ≥ 3 biological replicates; *P < .05 **P < .01, ***P < .001 by multiple unpaired *t*-test, FDR 5% against each native promoter; Related to Supplementary Fig. S1. Panels (A) and (C) were created in BioRender. Nissim, L. (2026) https://BioRender.com/sscn9yw.

Minimal promoters, a subclass of core promoters, are regulatory sequences typically located near the transcription start site (TSS) and control the efficiency of transcription initiation. Therefore, modulating minimal promoters provides an efficient methodology to tune gene expression independently of the enhancer [31, 55, 63–66]. To this end, we focused on systematic engineering of compact, modular, and tunable synthetic minimal promoters that yield predictable expression outcomes [31, 55, 63–66].

Minimal promoters comprise variable combinations of well-characterized motifs, such as the TATA box [54, 67], Initiator (Inr), downstream promoter element, downstream core element [68], and TFIIB recognition elements (BREu and BREd) [69, 70]. This architecture yields modular configurations in which the composition and arrangement of regulatory elements determine transcription initiation efficiency [5, 31, 55, 63–65, 67, 69, 71, 72]. Therefore, modifications to motif sequences and configurations enable tuning of promoter activity without altering the enhancer region [73]. For example, a super core promoter (SCP) that combines four canonical motif elements was rationally designed to enhance promoter activity in mammalian cells [54], and alterations to the TATA box motif in native yeast promoters enabled graded modulation of gene expression without increasing transcriptional noise [73]. Similar modularity strategies have been applied in other organisms.

Nevertheless, systematic optimization of minimal promoters tailored for synthetic promoters in mammalian cells is required to enable precise fine-tuning of gene expression, minimize basal transcriptional noise, and reduce encoding capacity requirements.

To this end, we established a systematic framework for engineering minimal promoters through targeted modifications of minimal promoter sequences, core element composition, and core element sequences. Our findings support a modular model of minimal promoter function in which *cis-*regulatory elements from different minimal promoters can be mixed, altered, added, or deleted to yield predictable expression outcomes. We further enhanced the activity of known minimal promoters by removing sequence redundancy, engineered tunable minimal promoters, generated candidate regulatory elements that span 7–8 bp, and designed artificial ${5^{\prime}}$ untranslated regions (${5^{\prime}}$ UTRs) that provide an additional modular layer of transgene regulation. This framework expands the synthetic promoter design capability and provides fine-tuning of gene expression, further advancing applications in biotechnology, biomedicine, and cellular engineering.

## Materials and methods

### Minimal promoter construction

Native Minimal promoter sequences were derived from previously characterized sequences, including miniCMVp [74], YB_TATAp [75], miniTKp [76], 90Kp [77], lateADEp [78, 79], and SCP [54]. The MEME suite was used to compare miniCMVp, YB_TATAp, miniTKp, and 90Kp for TATA box replacement [80]. Canonical elements were identified using the ElemeNT 2023 tool established by Tamar Juven-Gershon [81]. Other shared elements were identified by sequence alignment. Modifications involving block deletions of canonical or other shared elements were identified by sequence alignment or regions between motifs (Tables 3, 4, and 6).

### Plasmid construction

Plasmid assembly was performed using either Gibson assembly (NEB-E2621X) or T4 DNA ligation (NEB-M0202L), depending on the experimental design. Vector backbones were prepared by restriction digestion with the appropriate enzyme pairs (New England Biolabs) in CutSmart buffer, incubated overnight at 37°C. Digested fragments were resolved on 1% agarose gels and extracted using the QIAquick Gel Extraction Kit (Qiagen) following the manufacturer’s instructions. Insert fragments were amplified by polymerase chain reaction (PCR) using Phusion® HF PCR Master Mix (New England Biolabs), supplemented with 10 µl betaine (Sigma–Aldrich), 10 ng DNA template, and 20 µM sequence-specific primers. PCR was carried out for 28 cycles, and products were gel-extracted as described above. For ligation-based cloning, both insert and vector were digested under the same conditions and ligated at a 1:5 molar ratio using T4 DNA ligase (New England Biolabs, M0202S) at 16°C overnight. Gibson assemblies were performed using a 4:1 (vector:insert) volume ratio. Final constructs were transformed into HIT DH5α chemically competent *Escherichia coli* cells (Real Biotech) by incubation for 10 min on ice. Transformed cells were plated on LB-agar supplemented with ampicillin and incubated at 37°C overnight. Colonies were selected with colony PCR and cultured in 4 ml LB medium with ampicillin for plasmid amplification. Minipreps were performed using the NucleoSpin Plasmid EasyPure Kit (MAN-740727.250). Sequence verification was conducted by the National Center for Genomic Technologies (Givat Ram), and large-scale preparations were carried out using the QIAGEN Plasmid Plus Midi Kit (Qiagen).

Comparison of mKate2 output for native lateADEp-mKate2 and native YB_TATAp-mKate2 constructs, cloned in either forward (F) or reverse (R) orientations, is shown in (Extended Data Fig. 3). Neither orientation yielded a statistically significant difference, showing that these constructs can be used in both orientations.

Full sequences for all plasmids used in this study are detailed in GenBank format in the supplementary ZIP file “PlasmidSequences.zip.” Plasmids and plasmid sequences of the main 75 plasmids used in this study were also deposited to Addgene and can be found at:

https://www.addgene.org/browse/article/28275426/.

### Cell culture

Low-passage HEK293T cells (CRL-3216, ATCC), HeLa cells (CRM-CCL-2, ATCC), MDA-MB-231 (HTB-26, ATCC), SK-OV-3 (HTB-77, ATCC), aHDF (PCS-201-012, ATCC) and HPNE (CRL-4023, ATCC) were cultured in Dulbecco’s modified Eagle medium (DMEM; 01-055-1A, Biological Industries) supplemented with 10% fetal bovine serum (FBS; 04-007-1A, Biological Industries), 1% MEM non-essential amino acids (MEM/NEAA; 01-340-1B, Biological Industries), 1% sodium pyruvate (03-042-1B, Biological Industries), and 100 units/ml penicillin with 0.1 mg/ml streptomycin (03-031-1B, Biological Industries). HaCaT cells (300493, Cytion) and BEAS-2B (CRL-3588, ATCC) were maintained in RPMI-1640 medium (high glucose, glutamine-supplemented; Biological Industries) supplemented with 10% FBS (04-007-1A, Biological Industries), and 1% sodium pyruvate (03-042-1B, Biological Industries), and 100 units/ml penicillin with 0.1 mg/ml streptomycin (03-031-1B, Biological Industries). All cell cultures were maintained at 37°C in a humidified incubator with 5% CO₂.

### Lentivirus production

To ensure proper assessment of minimal promoter strength, lentiviruses were produced for each promoter variant in all experiments. Lentiviruses were produced in HEK293T cells grown in a six-well plate. A transfection mixture containing 6 µl SusFexin reagent (BG-TRSF), 100 µl Opti-MEM (31985-047, Thermo Fisher Scientific), and a three-plasmid DNA cocktail (0.5 µg pCMV-VSV-G (Addgene #8454), 0.5 µg psPAX2 (Addgene #12260), 1 µg lentiviral expression vector) was incubated at room temperature for 20 min. HEK293T suspension cells were diluted to 2.5 × 10⁶ cells/ml, and 0.5 ml (1.25 × 10⁶ cells) was added to each tube containing the transfection complexes. Mixtures were transferred into wells containing 1 ml of fresh medium and incubated at 37°C with 5% CO₂. After 18 h, medium was replaced with 2.5 ml fresh culture medium. Viral supernatant was collected 48 h post-transfection and filtered through a 0.45-µm syringe filter (Merck Millex, SLHVR33RS).

### Viral transduction

Viral supernatants were collected 48 h after lentivirus production and filtered through a 0.45-µm syringe filter. A volume of 0.5 ml viral supernatant was added to the target cell line (5 × 10⁵ cells/ml), and medium was added to a total volume of 2.5 ml. Hexadimethrine bromide (Sigma–Aldrich, Cat. Number 107689) was added to a final concentration of 8 µg/ml.

### RNA extraction

Cells were collected by centrifuging twice at a speed of 3000 rpm for 3 min and aspirating remaining medium. RNA extraction was performed using RNeasy Plus Mini Kit (Qiagen, Cat. Number: 74134).

### Reverse transcription (cDNA synthesis)

Complementary DNA (cDNA) was synthesized from purified total RNA using the qScript Reverse Transcriptase kit (Agentek, Cat. Number: 95049-100). Each 20 µl reaction mixture was prepared by combining 1 µg of RNA template, 4 µl of 5× qScript Reaction Mix, and 1 µl of qScript Reverse Transcriptase. The reaction was carried out in a thermal cycler using the following parameters: an initial incubation at 22°C for 5 min, followed by reverse transcription at 42°C for 30 min, and an enzyme inactivation step at 85°C for 5 min. Following the reaction, the resulting cDNA was diluted with nuclease-free water to achieve a final working concentration of 12.5 ng/µl.

### Quantitative PCR

Quantitative PCR (qPCR) was performed to evaluate the transcriptional activity of the engineered minimal promoters. Each 20 µl reaction consisted of 10 µl SYBR Green Master Mix (NEB, Cat. Number: NEB-M3003E), 2 µl of a forward and reverse primer mixture (20 µM), 6 µl of nuclease-free water, and 2 µl of cDNA template at a concentration of 12.5 ng/µl. The primers used were for either mKate2 (target gene) or GAPDH (housekeeping gene). All reactions were performed in technical triplicates to ensure reproducibility. CFX maestro program was used to analyze and quantify target gene expression (mKate2) using the ΔΔCt method. No-reverse-transcriptase (RT−) controls were performed for a subset of samples using the identical reaction composition and cycling conditions with nuclease-free water substituted for the reverse transcriptase, to confirm the absence of a genomic DNA contribution to the measured signal. qPCR was performed using primer sets specific to mKate2 (forward ${5^{\prime}}$-ACCAGCTTCATGTACGGCAG-${3^{\prime}}$, reverse ${5^{\prime}}$-TCTCTCCCATGTGAAGCCCT-${3^{\prime}}$; amplicon 99 bp) and the reference gene GAPDH (forward ${5^{\prime}}$-GAGAAGGCTGGGGCTCATTT-${3^{\prime}}$, reverse ${5^{\prime}}$-ATGACGAACATGGGGGCATC-${3^{\prime}}$; amplicon 83 bp).

### Enrichment of ${5^{\prime}}$-capped RNA using terminator ${5^{\prime}}$-phosphate-dependent exonuclease (TEX)

To enrich for primary transcripts, total RNA was treated with Terminator ${5^{\prime}}$-Phosphate-Dependent Exonuclease (TEX; Lucigen/Epicentre, Cat. Number: LGCTER51020-1kit). Two micrograms of total RNA was incubated with 1 Unit of TEX enzyme, 2 µl of Terminator 10× Reaction Buffer A and 0.5 µl of RiboGuard RNase Inhibitor for 60 min at 30°C in a total volume of 20 µl.

### RNA purification via lithium chloride precipitation

Following TEX treatment, RNA was purified using lithium chloride (LiCl). Thirty microliters of 7.5 M LiCl solution (AM9480, ThermoFisher) was added to the RNA sample with 30 µl of RNAse-free DDW, and incubated at −20°C for 30 min. The mixture was then centrifuged at max speed for 15 min at 4°C. The resulting RNA pellet was washed with 4°C 70% ethanol and centrifuged at max speed for 15 min at 4°C. Ethanol was carefully discarded and RNA pellet was resuspended with 17 µl RNAse free DDW and incubated on ice for 15 min.

### ${5^{\prime}}$ Rapid amplification of cDNA Ends (${5^{\prime}}$ RACE)

TSS were characterized using the ${5^{\prime}}$/${3^{\prime}}$ RACE Kit, 2nd Generation (Sigma–Aldrich, Cat. Number: 03353621001) according to the manufacturer’s experimental protocol for ${5^{\prime}}$ RACE. First-strand cDNA was synthesized from 0.2–2.0 µg of total RNA using an mKate2-specific primer (SP1: ${5^{\prime}}$-CCGTCTTCGTATGTGGTGACTC-${3^{\prime}}$) and Transcriptor Reverse Transcriptase at 55°C for 60 min, followed by inactivation at 85°C for 5 min. The cDNA was purified using the QIAquick PCR Purification Kit (QIAGEN, Cat. Number 28104) and ${3^{\prime}}$-dA-tailed using recombinant Terminal Transferase and dATP at 37°C for 20 min, followed by heat inactivation at 70°C for 10 min. Nested PCR amplification was performed in two successive rounds: the first round used an Oligo dT-anchor primer and a second mKate2-specific primer (SP2: ${5^{\prime}}$-GAAGCTGGTAGCCAGGATGTC-${3^{\prime}}$); the second round used the PCR anchor primer and a third mKate2-specific primer (SP3: ${5^{\prime}}$-GCCCTCGGATGTGCACTTG-${3^{\prime}}$). Amplification specificity was verified via agarose gel electrophoresis.

### Library preparation and next-generation sequencing

To enable high-resolution mapping of the TSS, the ${5^{\prime}}$ RACE PCR products were prepared for deep sequencing. Standard Illumina-compatible adapters were appended to the purified cDNA fragments via PCR amplification. The resulting sequencing libraries were then submitted to the next-generation sequencing (NGS) unit at the Hebrew University-Hadassah Medical School (Ein Kerem, Jerusalem) for processing. Seven and half microliters (7.5 µl) of the clean 1st PCR products underwent a secondary PCR to incorporate unique index sequences (N7XX and S5XX) for sample tagging, utilizing the Nextera XT Index Kit (Illumina, San Diego, CA, USA). Subsequently, 5 µl from each sample was combined, thoroughly mixed, and centrifuged. Finally, the DNA pool was purified with 1× AMPure XP beads and quantified using the Qubit® Fluorometer (Invitrogen). All DNA sample libraries were pooled to a 10 nM sample. Multiplex samples pool were loaded on NovaSeqX system (Illumina), using NovaSeq X Series 25B Reagent Kit (300 cycles) (cat# 20104706), with 161 cycles of paired-end sequencing.

### Data processing and analysis

Raw reads were processed with cutadapt, v4.7, in paired-end mode. Pairs where either of the reads ended with the adapter sequence were removed. Pairs where either of the reads did not start with the expected amplicon sequence, GAATTCGACCACGCGTATCGATGTCGAC for R1 reads and ACCGGTACCGGTGCCCTCCATGTACAGC for R2 reads, were removed. Remaining R1 reads were trimmed off the technical amplicon sequence start and the expected poly-T stretch, discarding pairs lacking those sequences. In order to allow for end-to-end alignment of pairs of reads, the reverse-complement of the amplicon sequence start and a poly-A stretch were trimmed off the ends of R2 reads. Finally, only pairs where both R1 and R2 reads were at least 60 bases long after the final trimming step were kept. Processed reads were aligned to the relevant reference for each sample. The reference sequence included the technical amplicon sequence start, then 30 Ts, then the specific sample promoter sequence and the common mKate2 sequence. Alignment was done with Bowtie2, v2.5.2, only to the forward strand, not allowing mixed alignments and discordant alignments, and allowing dovetail alignments. Other parameters were kept at default values.

Position of the TSS for each amplicon was determined by the first alignment position of R1 reads. Reads that aligned to a reference position before 59 were discarded, as the first 58 positions included the technical amplicon sequence start and poly-T stretch rather than the promoter sequence. The value 58 was then subtracted from the alignment position of each read in order to name first the nucleotide of the promoter as “1.” Data were organized in Excel, where each read was assigned to the position of its ${5^{\prime}}$-most nucleotide. For each promoter, the percentage of reads (counts) initiating at every position was calculated relative to the total read count across all positions. The position with the highest percentage of reads was designated the main TSS (+1).

### Cell Cycle Analysis using Hoechst 33342

To evaluate promoter activity across different stages of the cell cycle, transduced cells were subjected to live-cell DNA staining and flow cytometry analysis. Cells were first harvested via trypsinization, counted, and resuspended in phosphate buffered saline (PBS) to ensure a consistent population of ~10^6^ cells/ml. For DNA staining, a 0.01 mg/ml Hoechst 33342 (H3570, Thermo Fisher Scientific) solution was prepared from a 10 mg/ml initial stock. Two-hundred fifty microliters of resuspended cells were incubated with 400 µl of the Hoechst solution for 10 min at 37°C, while strictly protected from light. Following incubation, cells were centrifuged, and then resuspended in fresh PBS. Fluorescence was measured using CytoFLEX LX flow cytometer (Beckman Coulter, USA). A minimum of 10 000 live AND Hoechst 33342 positive cells were collected per sample.

### Flow cytometry

Flow cytometry was used to assess mKate2 levels after 5 days. Cells were washed with PBS, detached using 0.5 ml trypsin–EDTA, centrifuged, and resuspended in PBS. Fluorescence signals in all experiments (excluding cell cycle experiment, Supplementary Fig. S9C) were measured using a CytoFLEX S flow cytometer (Beckman Coulter, USA). A minimum of 25 000 live cells were collected per sample. Fluorescence signals in the cell cycle experiment (Supplementary Fig. S9C), were measured using a CytoFLEX LX flow cytometer (Beckman Coulter, USA). A minimum of 10 000 live AND Hoechst 33342 positive cells were collected per sample.

### Statistical and data analysis

Flow cytometry data were analyzed using Kaluza analysis software (Beckman Coulter). Graphs and statistical analyses were performed using GraphPad Prism 10.4.2 (GraphPad Software, San Diego, CA, USA), and additional calculations were conducted in Microsoft Excel. In Figs 1 –8, a multiple unpaired *t*-test, FDR 5% was applied to compare native promoters with altered variants in all datasets. In Supplementary Fig. S9A, One-way ANOVA followed by Tukey’s multiple-comparisons test was applied to compare between gDNA normalized RNA and gDNA normalized protein levels. In Supplementary Fig. S9B, One-way ANOVA followed by Tukey’s multiple-comparisons test was applied to compare Cq values between the RT+, RT−, NT, and DDW reactions. In Supplementary Fig. S9C, One-way ANOVA followed by Tukey’s multiple-comparisons test was used to determine changes between mKate2 levels in the cell cycle phases between the different minimal promoters. Datasets of weighted median fluorescence values/normalized weighted median fluorescence values were calculated using the formulas:

**Figure 2.**
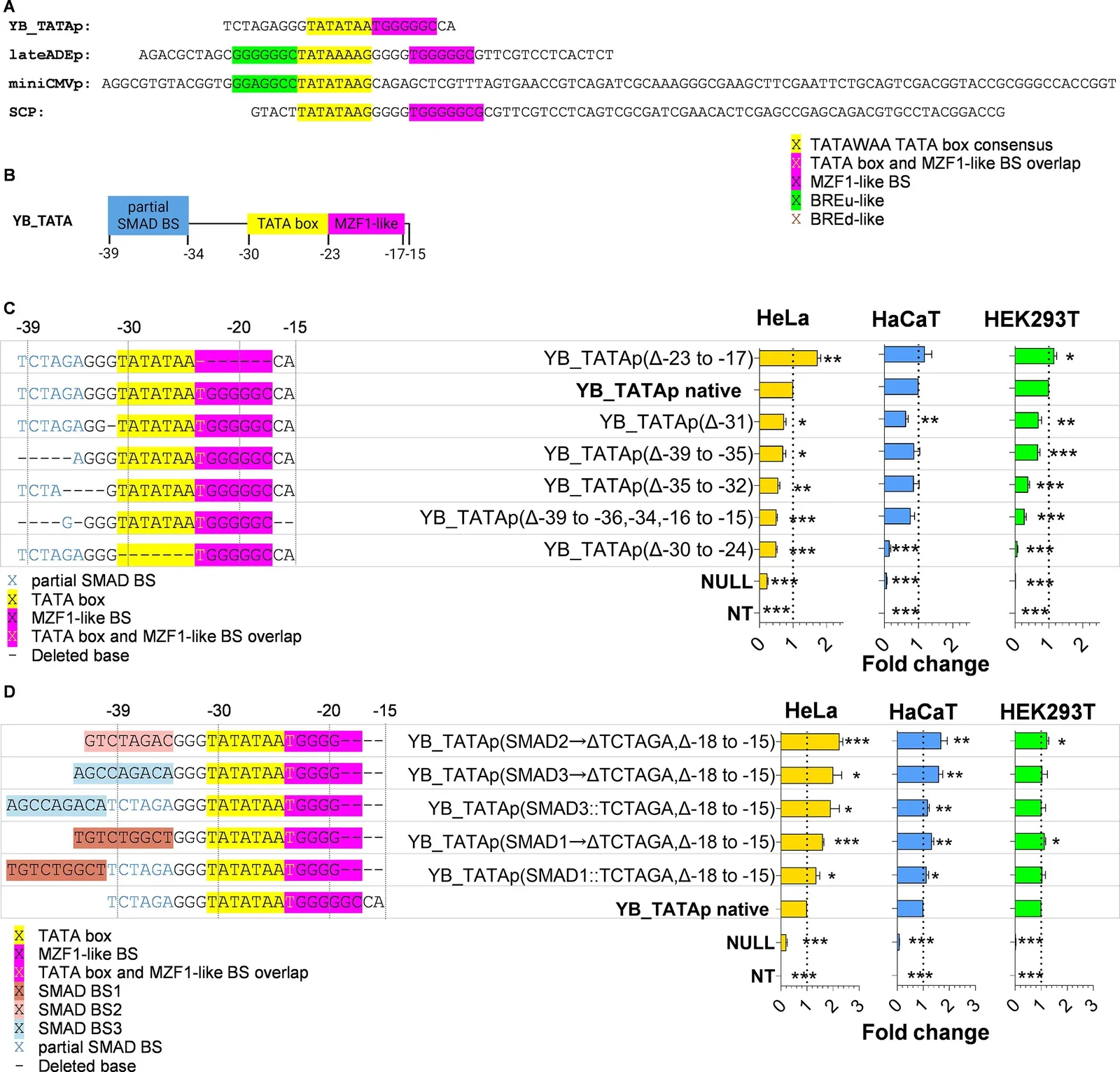
Deletion analysis and remodeling of the YB_TATA promoter reveals sequence features that fine-tune expression via repression or enhancement. (**A**) Four minimal promoter sequences with notable similarities in the core sequence composition as marked (Full sequences in Table 1). (**B**) Scheme illustrating the architecture of the YB_TATAp minimal promoter with elements identified by sequence analysis displayed to scale. (**C**) Deletion analysis of YB_TATAp. (**D**) The partial SMAD binding site (BS) motif (TCTAGA) was either replaced or placed downstream of various known full SMAD binding sites across HeLa, HaCaT, and HEK293T. For both panels, graphs show weighted median mKate2 fluorescence following systematic deletions or mutations, normalized to the native YB_TATAp, and presented in descending order of magnitude per HeLa cells. Full minimal promoter variation sequences in Table 4. − Denotes deleted nucleotides; NULL denotes the lack of a minimal promoter within the synthetic enhancer–reporter construct; and NT denotes non-treated, naïve cells. All minimal promoter variants are annotated relative to the native minimal promoter’s TSS (+1). Data are presented as the mean ± SEM; *n* ≥ 3 biological replicates; *P < .05 **P < .01, ***P < .001 by multiple unpaired *t*-test, FDR 5% against YB_TATAp native. Related to Supplementary Fig. S2. Panel (B) was created in BioRender. Nissim, L. (2026) https://BioRender.com/sscn9yw.

**Figure 3.**
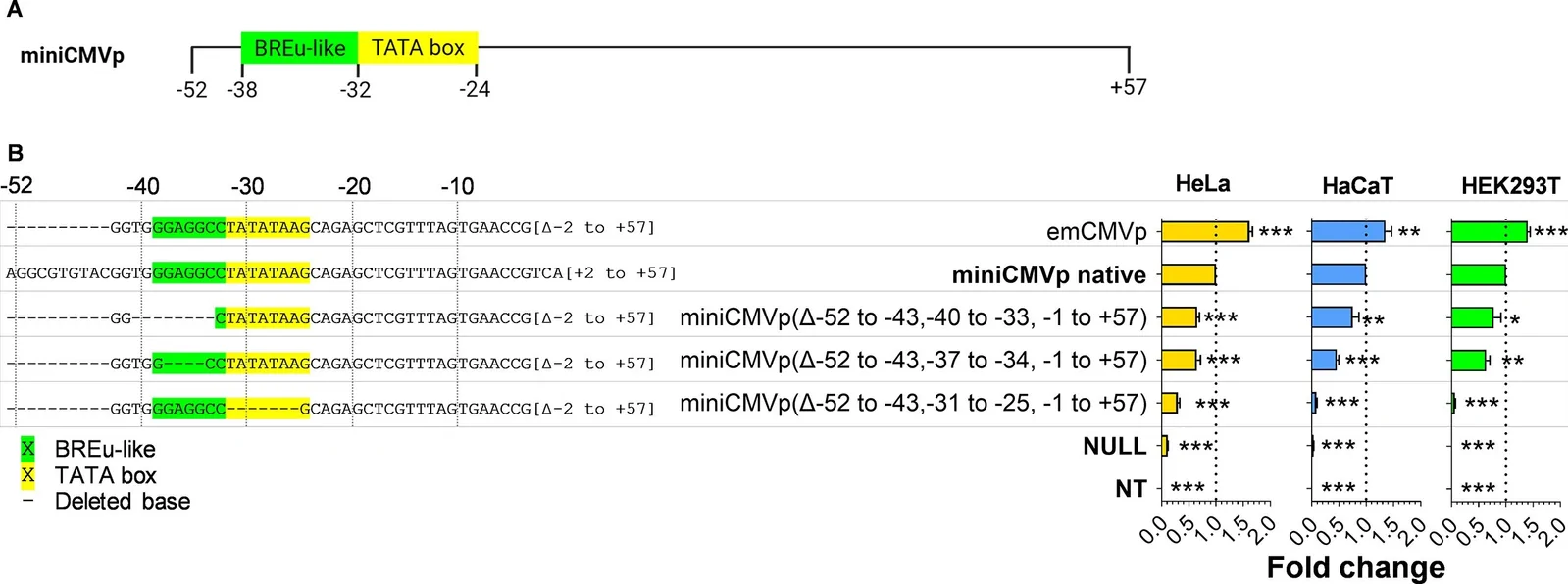
Deletions in the miniCMVp show sequence redundancy and maps promoter motif regions associated with increased or decreased output across three human cell types. (**A**) Schematic above graph illustrates architecture of the miniCMVp displaying the elements shared across promoters (Fig. 2A and Table 1). (**B**) Systematic deletion of identified elements within the miniCMVp reveals varied effects on expression, with enhanced activity following deletion of nucleotides upstream of the first identified element across HeLa, HaCaT, and HEK293T cells. Data are displayed as weighted median mKate2 normalized to the native miniCMVp, presented in descending order of magnitude per HeLa cells. Full minimal promoter variation sequences in Table 4. − Denotes deleted nucleotides; NULL denotes the lack of a minimal promoter within the synthetic enhancer–reporter construct; and NT denotes non-treated, naïve cells. All altered minimal promoter variants are annotated relative to the native minimal promoter’s TSS (+1). Square brackets indicate additional downstream nucleotides in the minimal promoter, with the position of non-displayed nucleotides shown in brackets (full sequences in Tables 1 and 4). Data are presented as the mean ± SEM; *n* ≥ 3 biological replicates; *P < .05 **P < .01, ***P < .001 by multiple unpaired *t*-test, FDR 5% against miniCMVp native. Related to Supplementary Fig. S3. Panel (A) was created in BioRender. Nissim, L. (2026) https://BioRender.com/sscn9yw.

**Figure 4.**
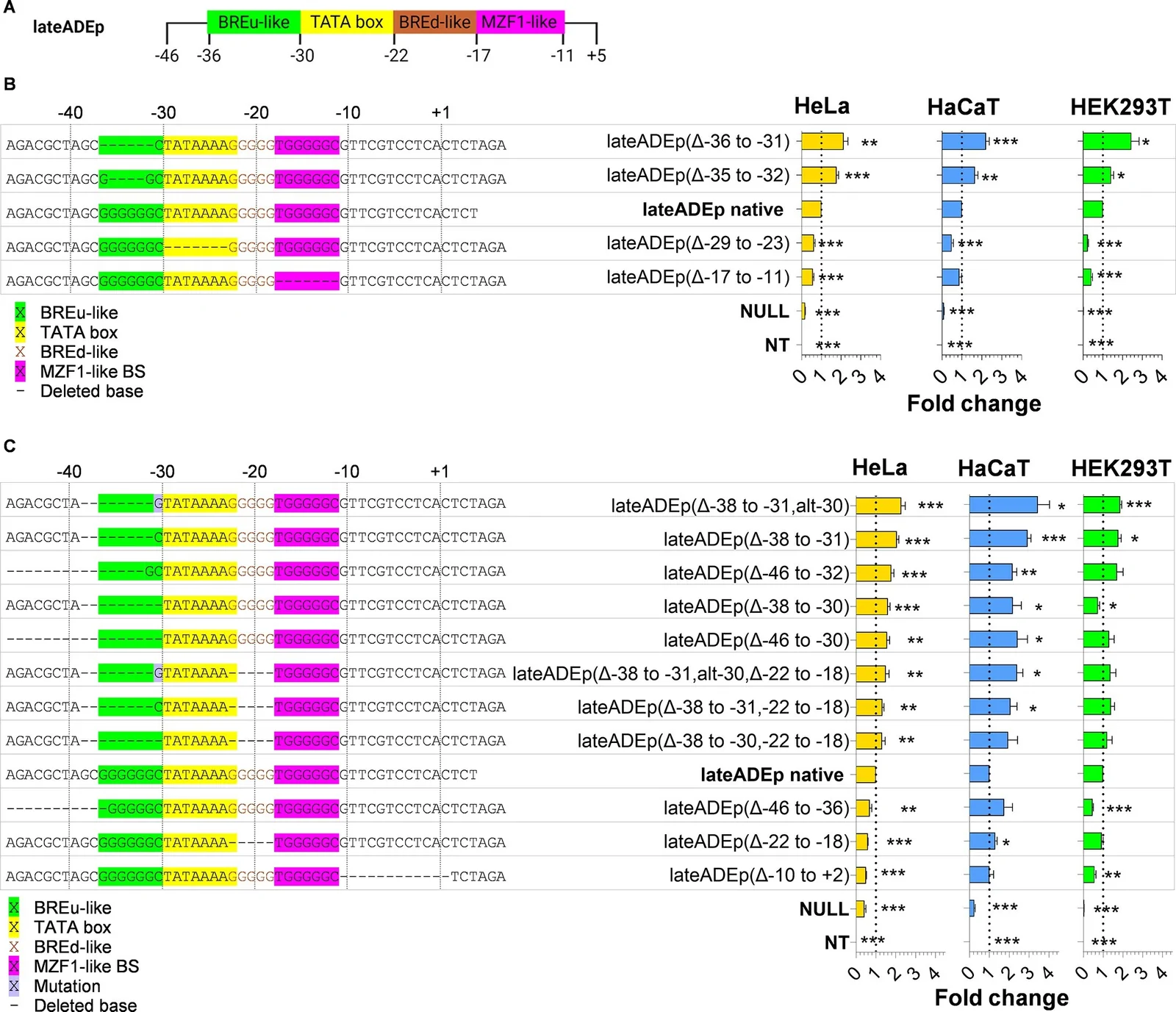
Targeted deletion analysis of the adenovirus major late promoter (lateADEp) reveals distinct sequence features that enhance or repress expression across three human cell types. (**A**) Schematic illustrating the architecture of the lateADEp displaying computationally and manually detected elements shared across promoters (Fig. 2A and Table 1). (**B**) Deletion analysis of lateADEp. (**C**) Combinatorial deletions and mutations were applied on the background of BREu-like motif region deletions. For both panels, graphs show weighted median mKate2 fluorescence following systematic deletions or mutations, normalized to the native lateADEp, and presented in descending order of magnitude per HeLa cells. Full minimal promoter variation sequences in Table 4. − Denotes deleted nucleotides; NULL denotes the lack of a minimal promoter within the synthetic enhancer–reporter construct; and NT denotes non-treated, naïve cells. All altered minimal promoter variants are annotated relative to the native minimal promoter’s TSS (+1). Data are presented as the mean ± SEM; *n* ≥ 3 biological replicates; *P < .05, **P < .01, ***P < .001 by multiple unpaired *t*-test, FDR 5% against lateADEp native. Related to Supplementary Fig. S4. Panel (A) was created in BioRender. Nissim, L. (2026) https://BioRender.com/sscn9yw.

**Figure 5.**
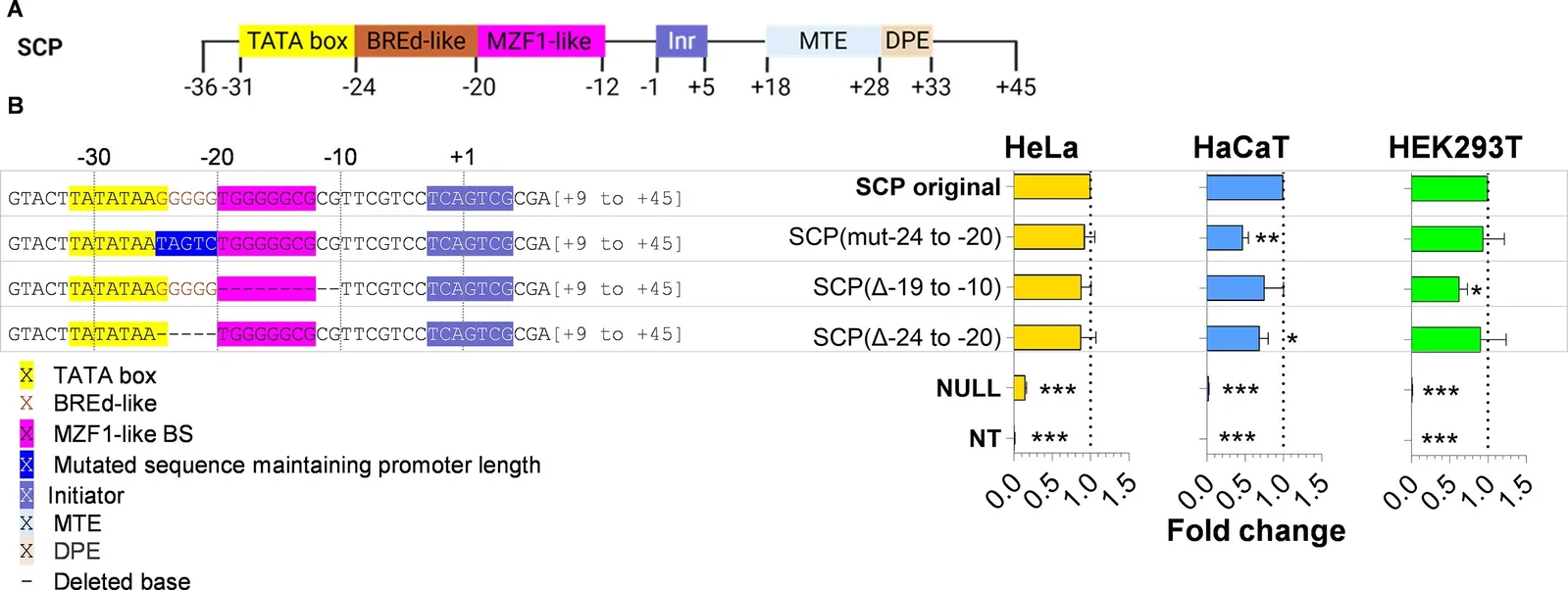
Modulatory mutational and deletion analysis of elements within the synthetic SCP results in minimal changes. (**A**) Schematic illustrating the architecture of the SCP displaying the elements shared across promoters (Fig. 2A and Table 1). (**B**) The graph shows the weighted median mKate2 fluorescence normalized to mKate2 from the native SCP across HeLa, HaCaT, and HEK293T cells, presented in descending order of magnitude per HeLa cells. Full minimal promoter variations sequences in Table 4. − Denotes deleted nucleotides; NULL denotes the lack of a minimal promoter within the synthetic enhancer–reporter construct; and NT denotes non-treated, naïve cells. All altered minimal promoter variants are annotated relative to the native minimal promoter’s TSS (+1). Square brackets indicate additional downstream nucleotides in the minimal promoter, with the position of non-displayed nucleotides shown in brackets (full sequences in Tables 1 and 4). Data are presented as the mean ± SEM; *n* = 3 biological replicates; *P < .05, **P < .01, ***P < .001 by multiple unpaired *t*-test, FDR 5% against SCP original. Related to Supplementary Fig. S5. Panel (A) was created in BioRender. Nissim, L. (2026) https://BioRender.com/sscn9yw.

**Figure 6.**
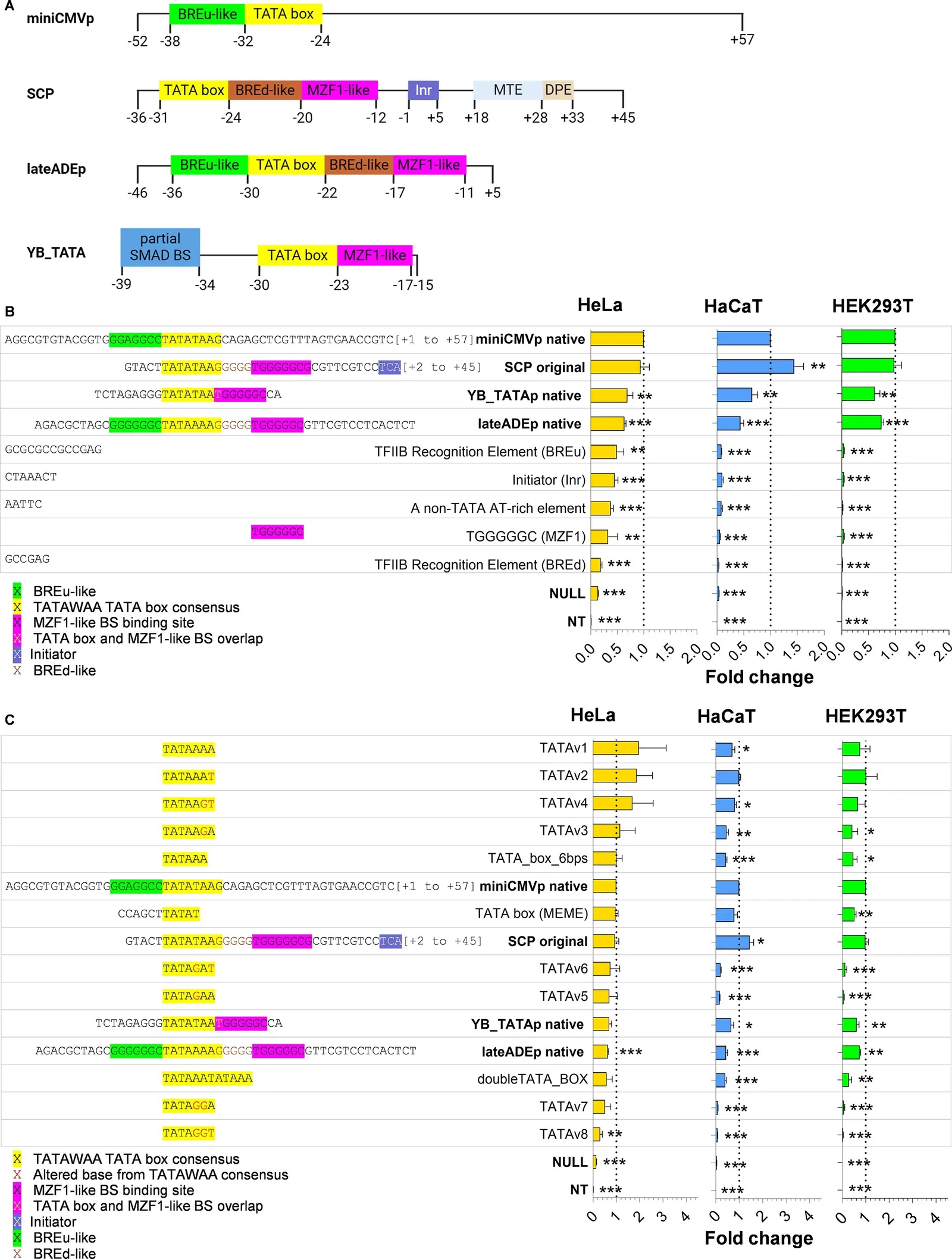
Isolating minimal promoter elements and systematic mutagenesis of TATA boxes reveals high-activity TATA box variants and gradient expression profiles. (**A**) Schematic overview of four similar minimal promoters from which individual elements were isolated and evaluated in the context of a synthetic enhancer-driven mKate2 reporter construct (enhancer sequence and vector backbone as in Fig. 1A and Table 2). (**B**) Constructs containing only a single element such as an Initiator (Inr), a non-TATA AT-rich sequence, or a TFIIB recognition element inserted in the minimal promoter position. (**C**) Constructs containing only a variation of a TATA box inserted in the minimal promoter position. Several TATA box variants were included (strategy for TATA box mutagenesis in Table 5). Both panels show weighted median mKate2 expression normalized to the native miniCMVp-mKate2 signal (reference for all data points) and presented in descending order of magnitude per HeLa cells. Full minimal promoter variations sequences in Table 4. Logic for TATA box variants in Table 5. NULL denotes the lack of a minimal promoter within the synthetic enhancer–reporter construct; NT denotes non-treated, naïve cells. All altered minimal promoter variants are annotated relative to the native minimal promoter’s TSS (+1). Square brackets indicate additional downstream nucleotides in the minimal promoter, with the position of non-displayed nucleotides shown in brackets (full sequences in Tables 1 and 4). Data are presented as the mean ± SEM; *n* = 3 biological replicates; *P < .05, **P < .01, ***P < .001 by multiple unpaired *t*-test, FDR 5% against miniCMVp native. Related to Supplementary Fig. S6. Panel (A) was created in BioRender. Nissim, L. (2026) https://BioRender.com/sscn9yw.

**Figure 7.**
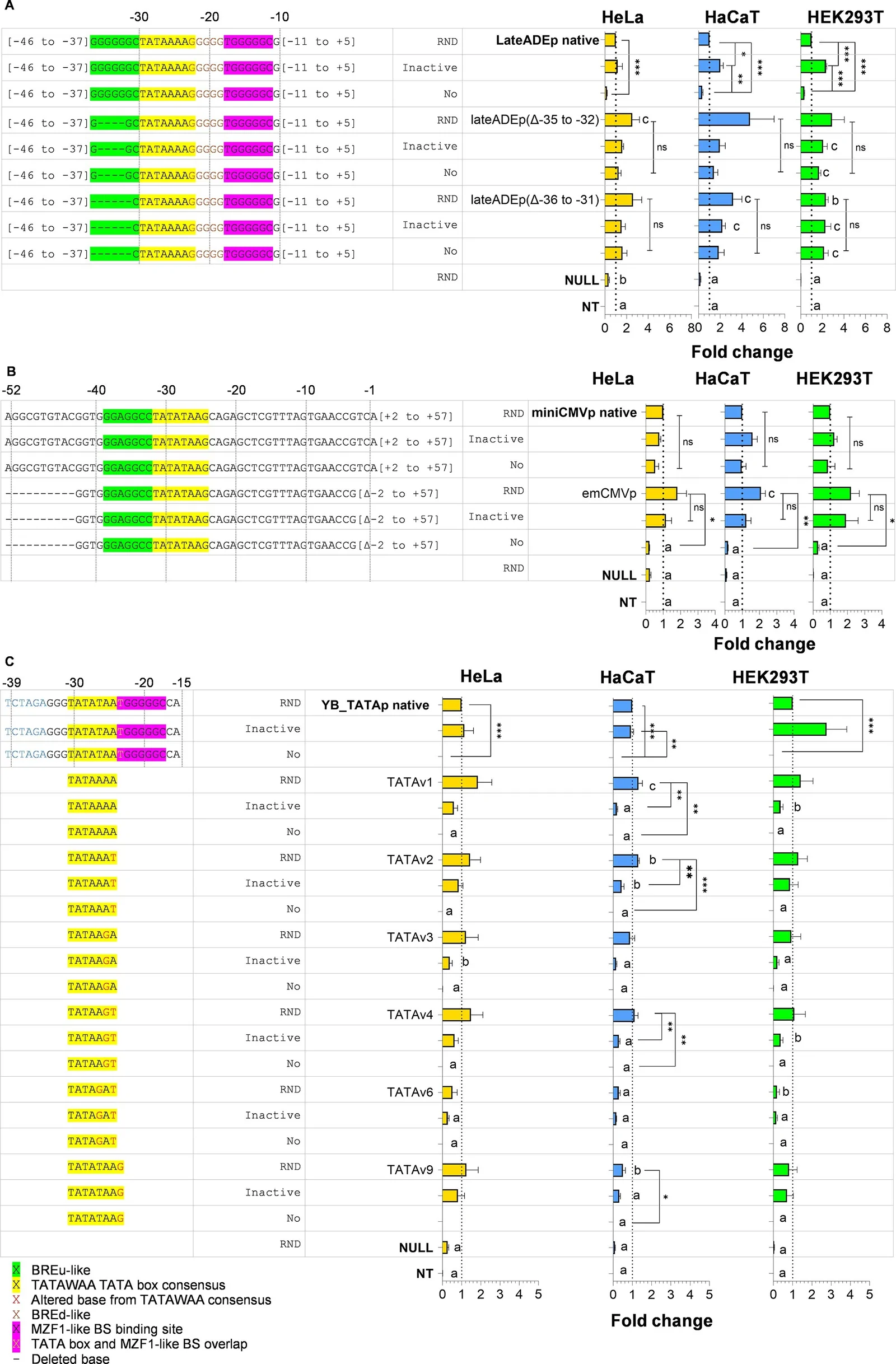
${5^{\prime}}$ UTR sequence modulates transcription in short versus long minimal promoter constructs. (**A**) Constructs containing previously designed lateADEp variants with different ${5^{\prime}}$UTR sequences. (**B**) Constructs containing a previously designed miniCMVp variant with different ${5^{\prime}}$UTR sequences. (**C**) Constructs containing only a TATA box inserted in the minimal promoter position with different ${5^{\prime}}$UTR sequences. Several TATA box variants were included (strategy for TATA box mutagenesis in Table 5). For all panels, graphs show weighted median mKate2 expression normalized to the corresponding native promoter-mKate2 signal (YB_TATAp, lateADEp, or miniCMVp) used as the reference. Data are grouped by promoter variants. Full ${5^{\prime}}$UTR and minimal promoter variations sequences in Tables 6 and 7. NULL denotes the lack of a minimal promoter within the synthetic enhancer–reporter construct; NT denotes non-treated, naïve cells. All altered minimal promoter variants are annotated relative to the native minimal promoter’s TSS (+1). Square brackets indicate additional downstream nucleotides in the minimal promoter, with the position of non-displayed nucleotides shown in brackets (full sequences in Tables 1, 3, and 7). Data are presented as the mean ± SEM; *n* = 3 biological replicates; *P < .05, **P < .01, ***P < .001; [c] *P* < .05 [b] *P* < .01, [a] *P* < .001, all analyzed by multiple unpaired *t*-test, FDR 5% against native (lateADEp, miniCMVp, YB_TATAp), or against variant-RND ${5^{\prime}}$UTR within each variant group. Related to Supplementary Fig. S7.

**Figure 8.**
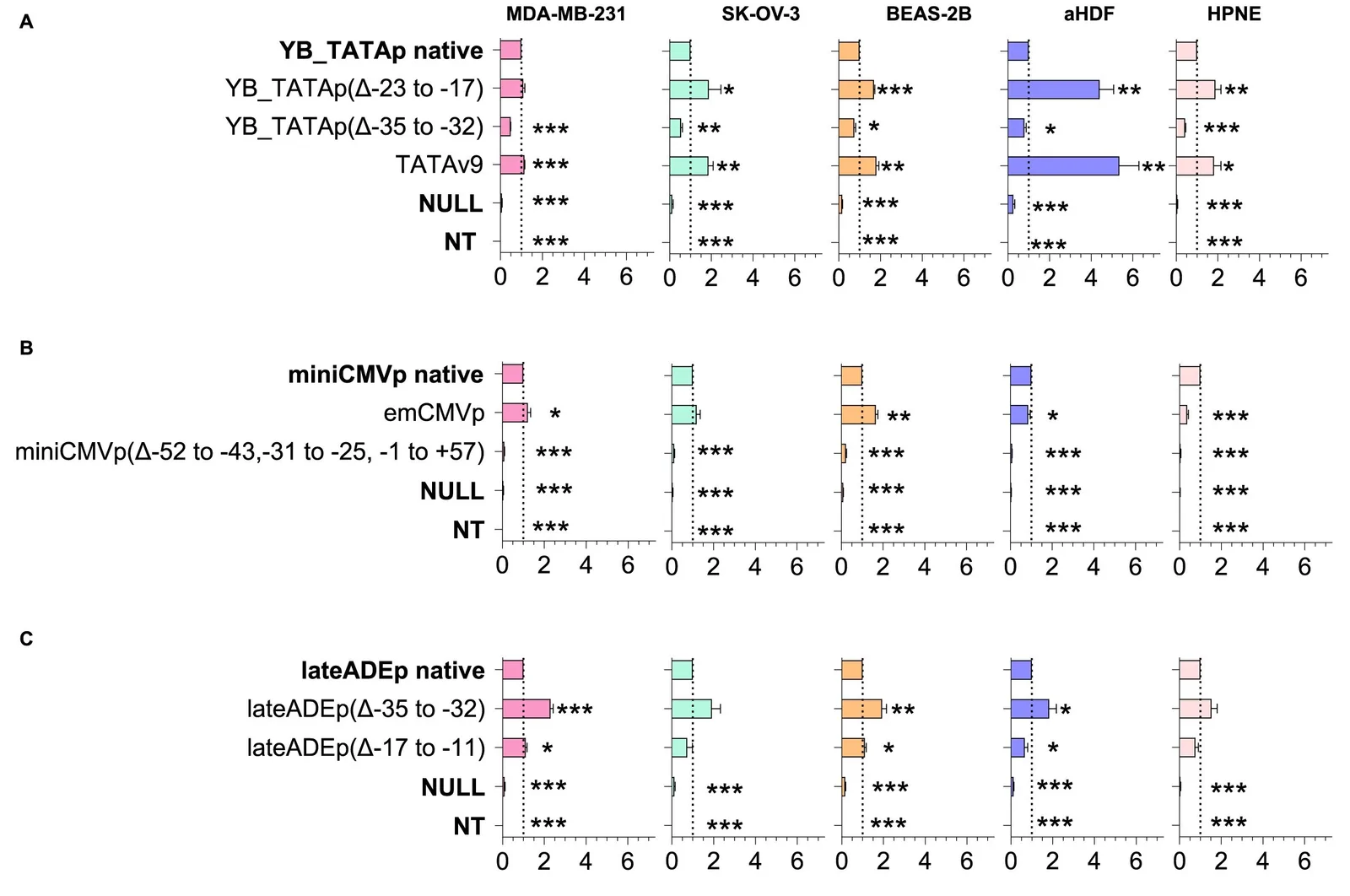
Minimal promoter activity patterns are broadly maintained across cell types. Representative minimal promoter variants were selected from the lateADEp, YB_TATAp, and miniCMVp groups to evaluate their functionality in five additional human cell types representing malignant and non-malignant cells from distinct tissues. For each architecture, two to three constructs were characterized across five additional human cell lines (MDA-MB-231, SK-OV-3, BEAS-2B, aHDF, and HPNE). (**A**) YB_TATAp variants, (**B**) miniCMVp variants, and (**C**) lateADEp variants. Graphs show weighted median mKate2 fluorescence, normalized to the corresponding native minimal promoter in each cell line. Full minimal promoter variation sequences are detailed in Tables 1, 4, and 7. NULL denotes the lack of a minimal promoter within the synthetic enhancer–reporter construct; NT denotes non-treated, naïve cells. Data are presented as the mean ± SEM; *n* = 3 biological replicates; *P < .05, **P < .01, ***P < .001 by multiple unpaired *t*-test, FDR 5% against each native. Related to Supplementary Fig. S8.

weighted median = (% positive fluorescence) × (median of the positive population). normalized weighted median = (weighted median of sample) ÷ (weighted median of native/control).

### Supplementary Fig. S9A calculations

#### Copy number normalization

To account for variations in plasmid copy number, RNA and protein expression levels were normalized to DNA abundance. Specifically, relative RNA (ΔΔCt) and protein (mKate2 weighted median) values were divided by the corresponding DNA ΔΔCt values.

#### Promoter activity normalization

Following copy number normalization, data were either presented as normalized to copy number values or normalized to the native minimal promoter. In the latter case, the expression levels of the engineered variants were divided by the values obtained for the native minimal promoter to reflect the fold change in activity.

All calculations and results are also available in the Raw Data file.

## Results

### Substitution analysis demonstrates context dependence of TATA box function in minimal promoters

The TATA box is a core promoter motif that recruits the TATA-binding protein, which mediates pre-initiation complex assembly [82, 83]. Although conserved in function, TATA box sequences vary across promoters, raising the question of whether these variations are interchangeable or context-dependent. To investigate this question, we examined four commonly used minimal promoters: the mini Cytomegalovirus promoter of a viral origin (miniCMVp) [74], the synthetic YB_TATAp [31], the viral mini thymidine kinase promoter (miniTKp) [76], and the human 90Kp [77] (Table 1). These minimal promoters were selected since miniCMVp and YB_TATAp contain TATA box sequences similar to the consensus sequence, whereas miniTKp contains a divergent TATA box. 90Kp, which lacks a TATA box, was included as a control (Table 1).

**Table 1.** Minimal promoter and control sequences

Plasmid	Promoter	Full sequence of minimal promoter
pMZ191	miniCMVp	AGGCGTGTACGGTGGGAGGCCTATATAAGCAGAGCTCGTTTAGTGAACCGTCAGATCGCAAAGGGCGAAGCTTCGAATTCTGCAGTCGACGGTACCGCGGGCCACCGGT
pMZ324 (R)/pYD2 (F)	YB_TATAp	TCTAGAGGGTATATAATGGGGGCCA
pMZ192	90Kp	CAGCAGCCAATCCCGGAAGACATCTATGTCAGGTGGTTTCTGGAAATCGAAAGTACCGGT
pMZ190	miniTKp	ACGCGTGTGGCCTCGAACACCGAGCGACCCTGCAGCGACCCGCTTAAAAACCGGT
pMZ323/pYD1 (F)	lateADEp	AGACGCTAGCGGGGGGCTATAAAAGGGGGTGGGGGCGTTCGTCCTCACTCT
pMZ313	SCP	GTACTTATATAAGGGGGTGGGGGCGCGTTCGTCCTCAGTCGCGATCGAACACTCGAGCCGAGCAGACGTGCCTACGGACCG
pMZ211	NULL	None

For this analysis, we first identified the TATA box consensus in each minimal promoter by the Multiple Em for Motif Elicitation analysis suite tool (MEME) [80] (Fig. 1B and C, and Supplementary Fig. S1A). We extended the identified consensus sequences by five downstream nucleotides in all minimal promoters, excluding miniTKp, which ends with a TATA-like element and was therefore extended by five upstream nucleotides. These extended regions were then reciprocally substituted among minimal promoters, generating a panel of chimeric constructs that were used to characterize transcriptional activity of each minimal promoter variant (Fig. 1D–G, Supplementary Fig. S1B–I, and Table 3). To maintain a consistent coordinate framework throughout the manuscript, we annotated the coordinates of each motif across all promoter variants relative to the TSS of the corresponding native promoter, designated as +1. The TSSs of native miniCMVp [74], YB_TATAp [31], and lateADEp [78, 79], used in this study, were determined experimentally (Extended Data Fig. 1), whereas the TSSs of the other native minimal promoters in this study were assigned from published reports [54, 76, 77].

As in all downstream experiments, we utilized lentiviral vectors in which the synthetic AHR:ARNT enhancer, a minimal promoter variant, and a randomly generated synthetic ${5^{\prime}}$ UTR (RND ${5^{\prime}}$ UTR) are sequentially encoded to regulate the expression of the mKate2 fluorescent reporter gene. This architecture was used as a basis for optimization since the AHR:ARNT binding sites drive strong and stable gene expression across a range of cell lines when coupled with native lateADEp and a downstream RND ${5^{\prime}}$UTR [84]. We separately transduced each cell sample with a lentiviral vector containing a single minimal promoter variant (Fig. 1A and Table 2). Subsequently, we estimated minimal promoter activity by flow cytometry measurements of weighted median mKate2 fluorescence (WM), which accounts for the percentage of fluorescence-positive cells and the median fluorescence intensity of this population (See the “Materials and methods” section for details of WM calculations) [85–87]. As initial models, we chose human cervical adenocarcinoma epithelial cells (HeLa), spontaneously immortalized adult human epidermal keratinocyte cells (HaCaT), and human embryonic kidney cells (HEK293T). Substitution analysis revealed distinct patterns and context-dependent effects. The control construct lacking a minimal promoter (NULL) did not produce detectable expression in any cell type, confirming the requirement for a core promoter for transcription initiation (Fig. 1D–G, Supplementary Fig. S1B–I, and Table 1). In miniCMVp, substitution with the consensus-like YB_TATAp TATA box enhanced expression only in HaCaT cells (Fig. 1D and Supplementary Fig. S1B and F). Replacement with the miniTKp TATA box significantly decreased expression in HEK293T cells, whereas HeLa and HaCaT were unaffected. Substitution with the 90Kp TATA box significantly reduced expression across all three cell types.

**Table 2.** Reporter backbone

Backbone region	Full sequence	Legend
AHR:ARNT enhancer region	**GGGGATCGCGTGACAACCC**ATC**GGGGATCGCGTGACAACCC**GCT**GGGGATCGCGTGACAACCC**TGA**GGGGATCGCGTGACAACCC**CAG**GGGGATCGCGTGACAACCC**	Bold text indicates Binding site of AHR:ARNT transcription factor complex.
5' UTR, KOZAK, mKate2 region	AGATCTGCGATCTAAGTAAGCTTGGCATTCCGGTACTGTTGGTAAACCAGCTCGCCACCATGGTGTCTAAGGGCGAAGAGCTGATTAAGGAGAACATGCACATGAAGCTGTACATGGAGGGCACCGTGAACAACCACCACTTCAAGTGCACATCCGAGGGCGAAGGCAAGCCCTACGAGGGCACCCAGACCATGAGAATCAAGGTGGTCGAGGGCGGCCCTCTCCCCTTCGCCTTCGACATCCTGGCTACCAGCTTCATGTACGGCAGCAAAACCTTCATCAACCACACCCAGGGCATCCCCGACTTCTTTAAGCAGTCCTTCCCTGAGGGCTTCACATGGGAGAGAGTCACCACATACGAAGACGGGGGCGTGCTGACCGCTACCCAGGACACCAGCCTCCAGGACGGCTGCCTCATCTACAACGTCAAGATCAGAGGGGTGAACTTCCCATCCAACGGCCCTGTGATGCAGAAGAAAACACTCGGCTGGGAGGCCTCCACCGAGATGCTGTACCCCGCTGACGGCGGCCTGGAAGGCAGAAGCGACATGGCCCTGAAGCTCGTGGGCGGGGGCCACCTGATCTGCAACTTGAAGACCACATACAGATCCAAGAAACCCGCTAAGAACCTCAAGATGCCCGGCGTCTACTATGTGGACAGAAGACTGGAAAGAATCAAGGAGGCCGACAAAGAGACCTACGTCGAGCAGCACGAGGTGGCTGTGGCCAGATACTGCGACCTCCCTAGCAAACTGGGGCACAAACTTAATTGA	
hUbCp-mKate2	GCGCCGGGTTTTGGCGCCTCCCGCGGGCGCCCCCCTCCTCACGGCGAGCGCTGCCACGTCAGACGAAGGGCGCAGCGAGCGTCCTGATCCTTCCGCCCGGACGCTCAGGACAGCGGCCCGCTGCTCATAAGACTCGGCCTTAGAACCCCAGTATCAGCAGAAGGACATTTTAGGACGGGACTTGGGTGACTCTAGGGCACTGGTTTTCTTTCCAGAGAGCGGAACAGGCGAGGAAAAGTAGTCCCTTCTCGGCGATTCTGCGGAGGGATCTCCGTGGGGCGGTGAACGCCGATGATTATATAAGGACGCGCCGGGTGTGGCACAGCTAGTTCCGTCGCAGCCGGGATTTGGGTCGCGGTTCTTGTTTGTGGATCGCTGTGATCGTCACTTGGTGAGTAGCGGGCTGCTGGGCTGGCCGGGGCTTTCGTGGCCGCCGGGCCGCTCGGTGGGACGGAAGCGTGTGGAGAGACCGCCAAGGGCTGTAGTCTGGGTCCGCGAGCAAGGTTGCCCTGAACTGGGGGTTGGGGGGAGCGCAGCAAAATGGCGGCTGTTCCCGAGTCTTGAATGGAAGACGCTTGTGAGGCGGGCTGTGAGGTCGTTGAAACAAGGTGGGGGGCATGGTGGGCGGCAAGAACCCAAGGTCTTGAGGCCTTCGCTAATGCGGGAAAGCTCTTATTCGGGTGAGATGGGCTGGGGCACCATCTGGGGACCCTGACGTGAAGTTTGTCACTGACTGGAGAACTCGGTTTGTCGTCTGTTGCGGGGGCGGCAGTTATGGCGGTGCCGTTGGGCAGTGCACCCGTACCTTTGGGAGCGCGCGCCCTCGTCGTGTCGTGACGTCACCCGTTCTGTTGGCTTATAATGCAGGGTGGGGCCACCTGCCGGTAGGTGTGCGGTAGGCTTTTCTCCGTCGCAGGACGCAGGGTTCGGGCCTAGGGTAGGCTCTCCTGAATCGACAGGCGCCGGACCTCTGGTGAGGGGAGGGATAAGTGAGGCGTCAGTTTCTTTGGTCGGTTTTATGTACCTATCTTCTTAAGTAGCTGAAGCTCCGGTTTTGAACTATGCGCTCGGGGTTGGCGAGTGTGTTTTGTGAAGTTTTTTAGGCACCTTTTGAAATGTAATCATTTGGGTCAATATGTAATTTTCAGTGTTAGACTAGTAAATTGTCCGCTAAATTCTGGCCGTTTTTGGCTTTTTTGTTAGACAGGATCCCCGGGTACCGGTCGCCACCATGGTGTCTAAGGGCGAAGAGCTGATTAAGGAGAACATGCACATGAAGCTGTACATGGAGGGCACCGTGAACAACCACCACTTCAAGTGCACATCCGAGGGCGAAGGCAAGCCCTACGAGGGCACCCAGACCATGAGAATCAAGGTGGTCGAGGGCGGCCCTCTCCCCTTCGCCTTCGACATCCTGGCTACCAGCTTCATGTACGGCAGCAAAACCTTCATCAACCACACCCAGGGCATCCCCGACTTCTTTAAGCAGTCCTTCCCTGAGGGCTTCACATGGGAGAGAGTCACCACATACGAAGACGGGGGCGTGCTGACCGCTACCCAGGACACCAGCCTCCAGGACGGCTGCCTCATCTACAACGTCAAGATCAGAGGGGTGAACTTCCCATCCAACGGCCCTGTGATGCAGAAGAAAACACTCGGCTGGGAGGCCTCCACCGAGATGCTGTACCCCGCTGACGGCGGCCTGGAAGGCAGAAGCGACATGGCCCTGAAGCTCGTGGGCGGGGGCCACCTGATCTGCAACTTGAAGACCACATACAGATCCAAGAAACCCGCTAAGAACCTCAAGATGCCCGGCGTCTACTATGTGGACAGAAGACTGGAAAGAATCAAGGAGGCCGACAAAGAGACCTACGTCGAGCAGCACGAGGTGGCTGTGGCCAGATACTGCGACCTCCCTAGCAAACTGGGGCACAAACTTAATTGA	

**Table 3.** Sequences of chimeric minimal promoters evaluated in Fig. 1 and Supplementary Fig. S1

plasmid	Minimal promoter name	Minimal promoter sequence
pMZ212	YB_TATA_miniCMVp_	TCTAGAGGGTATATAAGCAGAGCCA
pMZ213	miniTKp_YB_TATAp_	ACGCGTGTGGCCTCGAACACCGAGCGACCCTGCAGCGATATATAATGGGG
pMZ214	90Kp_YB_TATAp_	CAGCAGCCAATCCCGGAAGACATCTTATATAATGGGGTTTCTGGAAATCGAAAGT
pMZ215	miniCMVp_YB_TATAp_	AGGCGTGTACGGTGGGAGGCCTTATATAATGGGGGCTCGTTTAGTGAACCGTCAGATCGCAAAGGGCGAAGCTTCGAATTCTGCAGTCGACGGTACCGCGGGCC
pMZ216	YB_TATA_miniTKp_	TCTAGAGGGCCCGCTTAAAAGCCA
pMZ217	miniTKp_miniCMVp_	ACGCGTGTGGCCTCGAACACCGAGCGACCCTGCAGCGTATATAAGCAGA
pMZ218	90Kp_miniTKp_	CAGCAGCCAATCCCGGAAGACATCTCCCGCTTAAAATTTCTGGAAATCGAAAGT
pMZ219	miniCMVp_miniTKp_	AGGCGTGTACGGTGGGAGGCCTCCCGCTTAAAAGCTCGTTTAGTGAACCGTCAGATCGCAAAGGGCGAAGCTTCGAATTCTGCAGTCGACGGTACCGCGGGCC
pMZ220	YB_TATA_90Kp_	TCTAGAGGGATGTCAGGTGGGCCA
pMZ221	miniTKp_90Kp_	ACGCGTGTGGCCTCGAACACCGAGCGACCCTGCAGCGAATGTCAGGTGG
pMZ222	90Kp_miniCMVp_	CAGCAGCCAATCCCGGAAGACATCTATATAAGCAGATTTCTGGAAATCGAAAGT
pMZ223	miniCMVp_90Kp_	AGGCGTGTACGGTGGGAGGCCTATGTCAGGTGGGCTCGTTTAGTGAACCGTCAGATCGCAAAGGGCGAAGCTTCGAATTCTGCAGTCGACGGTACCGCGGGCC

Chimeric minimal promoters were generated by reciprocal substitution of TATA box regions and the corresponding region from 90Kp, which lacks a canonical TATA box. Construct names indicate the recipient minimal promoter, with the donor promoter shown in subscript. Sequences are presented in the 5′ to 3′ direction.

In YB_TATAp, substitution with the consensus-like miniCMVp TATA box significantly enhanced expression efficiency in HeLa and HEK293T cells, substitution with the miniTKp TATA box significantly lowered expression only in HaCaT and HEK293T cells, and the 90Kp TATA box significantly reduced activity across all three cell types (Fig. 1E and Supplementary Fig. S1C and G).

All miniTKp variants demonstrated low baseline promoter activity, likely due to dependence on specific enhancer context [76]. In this case, TATA box substitution did not significantly alter the absolute mKate2 expression levels in all three cell types (Fig. 1F and Supplementary Fig. S1D and H). Accordingly, the high normalized fold-change values should be evaluated with caution since at low baseline activity, minute differences in absolute values can yield large relative changes.

In 90Kp, substitution with the miniCMVp TATA attenuated promoter activity in HeLa and HaCaT cells (Fig. 1G and Supplementary Fig. S1E and I). Replacement with the YB_TATAp TATA box reduced expression selectively in HeLa cells, while activity in HaCaT and HEK293T remained comparable. Substitution with the miniTKp TATA box lowered expression across all three cell types.

Together, these results demonstrate that while the substitution approach provides a basis to modulate gene expression, TATA box motifs are not universally interchangeable, and their function depends on promoter architecture and cellular context.

### Unbiased minimal promoter engineering enables tunable gene expression

For unbiased identification of potential sequence features underlying minimal promoter function, we performed sequence alignment analysis of four potent minimal promoters with similar sequence composition: native miniCMVp, native YB_TATAp, native lateADEp [78, 79], and the SCP [54] (Fig. 2A and Table 1). We identified four different elements that appeared in at least two minimal promoters. First, we identified TATA box motifs in all minimal promoters. In lateADEp and miniCMVp, we found a GC-rich sequence resembling the upstream TFIIB recognition element (BREu), which is typically located upstream of the TATA box, and functions as a context-dependent transcriptional activator or repressor (herein termed the BREu-like motif). We also identified a 5-bp G-stretch located downstream of the TATA box in lateADEp and SCP (Fig. 2A), which is partially homologous to the known downstream TFIIB recognition element (herein termed the BREd-like motif), and functions similarly to BREu [83]. Another motif, MZF1-like (TGGGGGC), resembles the binding site of the myeloid zinc finger transcription factor (MZF1), which preferentially binds GC-rich sequences [88].

To evaluate the effects of modifying each identified element on output levels, we first performed a systematic deletion analysis in the YB_TATAp. Within this promoter, we also identified a partial SMAD binding site (TCTAGA) [89], which was therefore evaluated as well (Fig. 2B). The YB_TATAp(Δ−23 to −17) variant, in which the MZF1-like element was deleted, significantly enhanced mKate2 expression in HeLa cells. Deletions of other elements predominantly attenuated promoter activity, with partially distinct patterns between the cell types (Fig. 2C and Supplementary Fig. S2A). Additionally, reconstruction or addition of SMAD binding sites enhanced promoter activity in HeLa and HaCaT cells (Fig. 2D and Supplementary Fig. S2B).

We further extended the deletion approach to the 109-bp miniCMVp, including the removal of identified elements and of regions that were not highlighted in the sequence alignment analysis (Fig. 3A and B, and Supplementary Fig. S3). Deletion of 69 nucleotides from regions flanking the identified BREu-like and TATA box elements generated a truncated 40-bp minimal promoter. Surprisingly, this deletion enhanced reporter expression in all three cell types, resulting in a compact Enhanced miniCMVp (emCMVp). In contrast, deletion of the TATA box element significantly reduced expression. Finally, partial or full removal of the BREu-like motif site significantly reduced output expression in all three cell lines.

In the lateADEp, targeted deletion of the BREu-like motif (Δ−35 to −32 or Δ−36 to −31) provided a significant increase in relative reporter levels across all three cell types (Fig. 4A and B, and Supplementary Fig. S4A). Deletions of individual elements, such as MZF1-like (Δ−17 to −11) or the TATA box (Δ−29 to −23), predominantly attenuated relative reporter output in all three cell types (Fig. 4B and Supplementary Fig. S4A). Next, we examined full or partial deletions of multiple sequences, including the BREu-like motif, the 10-bp upstream of the BREu-like motif, and the BREd (Fig. 4A and B, and Supplementary Fig. S4A). Deletions of the BREu-like motif alone, or together with its upstream sequence, predominantly enhanced relative minimal promoter activity across all three cell types. Finally, deletion of the BREd-like motif alone reduced expression in HeLa cells, and deletion of 12 nucleotides downstream of the MZF1-like binding sites significantly attenuated promoter activity in HeLa and HEK293T cells (Fig. 4C and Supplementary Fig. S4B).

Although the SCP minimal promoter is highly optimized for maximal activity [54], we next performed modular deletion and mutation analysis targeting non-canonical elements that had not been previously examined, including the MZF1-like binding site and the BREd-like motif. In HeLa cells, these modifications did not affect expression, and overall promoter strength was preserved. In HaCaT cells, mutating (mut −24 to −20) or deleting (Δ−24 to −20) the BREd-like motif attenuated expression, whereas deleting positions −19 to −10 had no effect. In HEK293T cells, attenuated expression was observed only following deletion of the MZF1-like site (Δ−19 to −10), while mutation (mut−24 to −20) or deletion (Δ−24 to −20) of the BREd-like motif did not alter expression (Fig. 5A and B, and Supplementary Fig. S5).

Collectively, these results further indicate that sequence alignment can highlight shared minimal promoter sequences as candidate regulatory elements [90–92]. Together with canonical regulatory motifs, these elements may function as modular components that support the context-dependent tuning of minimal promoter activity. Notably, deletions can yield shorter minimal promoters with enhanced or attenuated activity, further demonstrating the potential of systematic minimal promoter engineering for optimized gene expression in mammalian cells.

### Single-element analyses provide strong and tunable candidate regulatory elements

We next examined the functionality of individual canonical and sequence-alignment-derived regulatory elements by systematically testing constructs containing only the consensus sequence or variations of a single element (Fig. 6 and Tables 4 and 5). Specifically, we tested BREu, Inr, MZF1, BREd, and a non-TATA AT-rich element (AATTC) (Fig. 6B), as well as TATA box variations (Fig. 6C). Single-element constructs that did not contain a TATA box or variations of its sequence produced no detectable signal in HaCaT and HEK293T, comparable to the NULL control. In HeLa cells, these elements generated graded expression ranging from levels comparable to the native miniCMVp to the NULL control, enabling fine-tuned gene expression in these cells (Fig. 6B and Supplementary Fig. S6A). These findings are consistent with the known combinatorial and context-dependent function of regulatory motifs [63, 65].

**Table 4.** Sequences of plasmids used in Figs 2–6 and 8, and Supplementary Figs S2–S6 and S8 and S9

plasmid	Minimal promoter name	Minimal promoter sequence
pMZ200	TATA box (MEME)	CCAGCTTATAT
pMZ206	TATA_box_6bps	TATAAA
pMZ207	Initiator (Inr)	CTAAACT
pMZ208	TFIIB Recognition Element (BRE)_u	GCGCGCCGCCGAG
pMZ209	TFIIB Recognition Element (BRE)_d	GCCGAG
pMZ210	AT rich element	AATTC
pMZ248	emCMVp	———-GGTGGGAGGCCTATATAAGCAGAGCTCGTTTAGTGAACCG[Δ-2 to + 57]
pMZ254	YB_TATAp(SMAD2→ΔTCTAGA,Δ-18 to -15)	GTCTAGACGGGTATATAATGGGG—-
pMZ255	YB_TATAp(SMAD3::TCTAGA,Δ-18 to -15)	AGCCAGACATCTAGAGGGTATATAATGGGG—-
pMZ256	YB_TATAp(SMAD3→ΔTCTAGA,Δ-18 to -15)	AGCCAGACAGGGTATATAATGGGG—-
pMZ257	YB_TATAp(SMAD1::TCTAGA,Δ-18 to -15)	TGTCTGGCTTCTAGAGGGTATATAATGGGG—-
pMZ258	YB_TATAp(SMAD1→ΔTCTAGA,Δ-18 to -15)	TGTCTGGCTGGGTATATAATGGGG—-
pMZ275	miniCMVp(Δ-52 to -43,-37 to -34, -1 to +57)	———-GGTGG—-CCTATATAAGCAGAGCTCGTTTAGTGAACCGTC[Δ-1 to + 57]
pMZ276	miniCMVp(Δ-52 to -43,-31 to -25, -1 to +57)	———-GGTGGGAGGCC——-GCAGAGCTCGTTTAGTGAACCGTC[Δ-1 to + 57]
pMZ278	miniCMVp(Δ-52 to -43,-40 to -33, -1 to +57)	———-GG——–CTATATAAGCAGAGCTCGTTTAGTGAACCGTC[Δ-1 to + 57]
pMZ279	YB_TATAp(Δ-30 to -24)	TCTAGAGGG——-TGGGGGCCA
pMZ280	YB_TATAp(Δ-23 to -17)	TCTAGAGGGTATATAA——-CA
pMZ281	YB_TATAp(Δ-39 to -35)	—–AGGGTATATAATGGGGGCCA
pMZ282	YB_TATAp(Δ-35 to -32)	TCTA—-GTATATAATGGGGGCCA
pMZ285	lateADEp(Δ-29 to -23)	AGACGCTAGCGGGGGGC——-GGGGGTGGGGGCGTTCGTCCTCACTCTAGA
pMZ286	lateADEp(Δ-35 to -32)	AGACGCTAGCG—-GCTATAAAAGGGGGTGGGGGCGTTCGTCCTCACTCTAGA
pMZ288	lateADEp(Δ-17 to -11)	AGACGCTAGCGGGGGGCTATAAAAGGGGG——-GTTCGTCCTCACTCTAGA
pMZ289	lateADEp(Δ-36 to -31)	AGACGCTAGC——CTATAAAAGGGGGTGGGGGCGTTCGTCCTCACTCTAGA
pMZ291	YB_TATAp(Δ-39 to -36,-34,-16 to -15)	—-G-GGGTATATAATGGGGGC–
pMZ292	YB_TATAp(Δ-31)	TCTAGAGG-TATATAATGGGGGCCA
pMZ300	lateADEp(Δ-38 to -31,alt-30)	AGACGCTA——–GTATAAAAGGGGGTGGGGGCGTTCGTCCTCACTCTAGA
pMZ302	lateADEp(Δ-38 to -30)	AGACGCTA———TATAAAAGGGGGTGGGGGCGTTCGTCCTCACTCTAGA
pMZ303	lateADEp(Δ-38 to -31)	AGACGCTA——–CTATAAAAGGGGGTGGGGGCGTTCGTCCTCACTCTAGA
pMZ305	lateADEp(Δ-22 to -18)	AGACGCTAGCGGGGGGCTATAAAA—–TGGGGGCGTTCGTCCTCACTCTAGA
pMZ306	lateADEp(Δ-38 to -30,-22 to -18)	AGACGCTA———TATAAAA—–TGGGGGCGTTCGTCCTCACTCTAGA
pMZ307	lateADEp(Δ-38 to -31,-22 to -18)	AGACGCTA——–CTATAAAA—–TGGGGGCGTTCGTCCTCACTCTAGA
pMZ308	lateADEp(Δ-38 to -31,alt-30,Δ-22 to -18)	AGACGCTA——–GTATAAAA—–TGGGGGCGTTCGTCCTCACTCTAGA
pMZ309	lateADEp(Δ-10 to +2)	AGACGCTAGCGGGGGGCTATAAAAGGGGGTGGGGGC————TCTAGA
pMZ310	lateADEp(Δ-46 to -36)	———–GGGGGCTATAAAAGGGGGTGGGGGCGTTCGTCCTCACTCTAGA
pMZ311	lateADEp(Δ-46 to -32)	—————GCTATAAAAGGGGGTGGGGGCGTTCGTCCTCACTCTAGA
pMZ312	lateADEp(Δ-46 to -30)	—————–TATAAAAGGGGGTGGGGGCGTTCGTCCTCACTCTAGA
pMZ332	TATAv1	TATAAAA
pMZ333	TATAv2	TATAAAT
pMZ334	TATAv3	TATAAGA
pMZ335	TATAv4	TATAAGT
pMZ336	TATAv5	TATAGAA
pMZ337	TATAv6	TATAGAT
pMZ338	TATAv7	TATAGGA
pMZ339	TATAv8	TATAGGT
pMZ340	doubleTATA_BOX	TATAAATATAAA
pMZ341	TGGGGGC (MZF1)	TGGGGGC
pMZ343	SCP(mut-24 to -20)	GTACTTATATAATAGTCTGGGGGCGCGTTCGTCCTCAGTCGCGATCGAACACTCGAGCCGAGCAGACGTGCCTACGGACCG
pMZ344	SCP(Δ-24 to -20)	GTACTTATATAA—–TGGGGGCGCGTTCGTCCTCAGTCGCGATCGAACACTCGAGCCGAGCAGACGTGCCTACGGACCG
pMZ345	SCP(Δ-19 to -10)	GTACTTATATAAGGGGG———-TTCGTCCTCAGTCGCGATCGAACACTCGAGCCGAGCAGACGTGCCTACGGACCG

**Table 5.** TATA box mutation strategy

	T	A	T	A	R	R	W
TATAv1	T	A	T	A	A	A	A
TATAv2	T	A	T	A	A	A	T
TATAv3	T	A	T	A	A	G	A
TATAv4	T	A	T	A	A	G	T
TATAv5	T	A	T	A	G	A	A
TATAv6	T	A	T	A	G	A	T
TATAv7	T	A	T	A	G	G	A
TATAv8	T	A	T	A	G	G	T

Furthermore, several TATA box variants enabled expression levels comparable to those of an entire native minimal promoter (Fig. 6C and Supplementary Fig. S6B). In HeLa cells, TATAv1 to TATAv6, a 6-bp TATA box, and a TATA box sequence representing the TATA box consensus derived from sequence-alignment analysis of the known minimal promoters (MEME) drove expression comparable to that of the miniCMVp. In HaCaT cells, TATAv2 and the TATA box (MEME) drove expressions comparable to miniCMVp. In HEK293T cells, TATAv1, TATAv2, and TATAv4 generated expression comparable to that of miniCMVp. Some variants also showed a subtle cell-type-dependent relative activity. For example, TATAv5 and TATAv6 exhibited unchanged expression in HeLa cells but reduced expression in HaCaT and HEK293T cells, indicating that certain TATA box variants might be more effective in specific cellular contexts.

These results demonstrate the feasibility of strong and tunable candidate regulatory elements spanning as little as 7 bp that can activate efficient transcription when combined with an appropriate ${5^{\prime}}$UTR, taking into account their context and cell-type-dependent activity.

### ${5^{\prime}}$ UTR variants provide an additional layer for tuning gene expression

The ${5^{\prime}}$ UTR plays a key role in gene expression, including the regulation of transcription initiation, translation initiation, and mRNA stability [93–95]. In this study, we used a randomly generated synthetic ${5^{\prime}}$ UTR sequence comprising 53 bp (RND ${5^{\prime}}$UTR) (Table 6). We analyzed the RND ${5^{\prime}}$UTR using the Juven-Gershon elemeNT2023 tool [81] and identified known minimal promoter regulatory elements, some of which potentially interact with motifs present in YB_TATAp, miniCMVp, and lateADEp (Extended Data Table 1 and Extended Data Fig. 2). Therefore, the RND ${5^{\prime}}$UTR may functionally support minimal promoter activity. To examine this hypothesis, we used the elemeNT2023 tool to design an additional synthetic ${5^{\prime}}$ UTR that lacks any known regulatory elements (Inactive ${5^{\prime}}$UTR) (Table 6). We then compared the output levels generated by native and minimal promoter variants, including the strong candidate regulatory elements, when combined with the RND ${5^{\prime}}$UTR, Inactive ${5^{\prime}}$UTR, or no ${5^{\prime}}$UTR (Fig. 7 and Table 7).

**Table 6.** Sequences of ${5^{\prime}}$UTRs used in Fig. 7 and Supplementary Fig. S7

5’ UTR name	Sequence
RND ${5^{\prime}}$UTR	AGATCTGCGATCTAAGTAAGCTTGGCATTCCGGTACTGTTGGTAAACCAGCTC
Inactive ${5^{\prime}}$UTR	AATTGGTGCCGCGAAGAAGCCTGGTTAAGGAGAACTCTCGGCGTAA

**Table 7.** Sequences of plasmids used in Fig. 7 and Supplementary Fig. S7

plasmid	Minimal promoter name	Sequence	5’UTR name
pMZ349	TATAv1	TATAAAA	None
pMZ350	TATAv2	TATAAAT	None
pMZ351	TATAv3	TATAAGA	None
pMZ352	TATAv4	TATAAGT	None
pMZ353	emCMVp	———-GGTGGGAGGCCTATATAAGCAGAGCTCGTTTAGTGAACCG[Δ-2 to + 57]	None
pMZ355	TATAv6	TATAGAT	None
pMZ356	lateADEp(Δ-35 to -32)	AGACGCTAGCG—-GCTATAAAAGGGGGTGGGGGCGTTCGTCCTCACTCTAGA	None
pMZ357	lateADEp(Δ-36 to -31)	AGACGCTAGC——CTATAAAAGGGGGTGGGGGCGTTCGTCCTCACTCTAGA	None
pMZ358	TATAv1	TATAAAA	Inactive
pMZ359	TATAv2	TATAAAT	Inactive
pMZ360	TATAv3	TATAAGA	Inactive
pMZ361	TATAv4	TATAAGT	Inactive
pMZ362	emCMVp	———-GGTGGGAGGCCTATATAAGCAGAGCTCGTTTAGTGAACCG[Δ-2 to + 57]	Inactive
pMZ364	TATAv6	TATAGAT	Inactive
pMZ365	lateADEp(Δ-35 to -32)	AGACGCTAGCG—-GCTATAAAAGGGGGTGGGGGCGTTCGTCCTCACTCTAGA	Inactive
pMZ366	lateADEp(Δ-36 to -31)	AGACGCTAGC——CTATAAAAGGGGGTGGGGGCGTTCGTCCTCACTCTAGA	Inactive
pMZ367	TATAv9	TATATAAG	RND
pMZ368	TATAv9	TATATAAG	None
pMZ369	TATAv9	TATATAAG	Inactive
pMZ370	YB_TATA	TCTAGAGGGTATATAATGGGGGCCA	None
pMZ371	miniCMVp	AGGCGTGTACGGTGGGAGGCCTATATAAGCAGAGCTCGTTTAGTGAACCGTCAGATCGCAAAGGGCGAAGCTTCGAATTCTGCAGTCGACGGTACCGCGGGCC	None
pMZ372	lateADEp	AGACGCTAGCGGGGGGCTATAAAAGGGGGTGGGGGCGTTCGTCCTCACTCTAGA	None
pMZ373	YB_TATA	TCTAGAGGGTATATAATGGGGGCCA	Inactive
pMZ374	miniCMVp	AGGCGTGTACGGTGGGAGGCCTATATAAGCAGAGCTCGTTTAGTGAACCGTCAGATCGCAAAGGGCGAAGCTTCGAATTCTGCAGTCGACGGTACCGCGGGCC	Inactive
pMZ375	lateADEp	AGACGCTAGCGGGGGGCTATAAAAGGGGGTGGGGGCGTTCGTCCTCACTCTAGA	Inactive

Surprisingly, the native lateADEp generated higher output when combined with the Inactive ${5^{\prime}}$UTR than with the RND ${5^{\prime}}$UTR in HaCaT and HEK293T cells, whereas removal of the ${5^{\prime}}$UTR resulted in markedly lower expression in all three cell types. Yet, the lateADEp variants in which the BREu-like element was fully or partially deleted demonstrated comparable performance that was independent of the ${5^{\prime}}$UTR (Fig. 7A and Supplementary Fig. S7A), suggesting that BREu-like element may interact with the ${5^{\prime}}$UTR. In comparison, the ${5^{\prime}}$UTR had a minimal effect on the native miniCMVp (Fig. 7B and Supplementary Fig. S7B). When combined with the compact emCMVp variation in which most of the sequence downstream to the TSS was deleted, both the Inactive ${5^{\prime}}$UTR and the RND ${5^{\prime}}$UTR demonstrated comparable performance. Yet, removal of the ${5^{\prime}}$ UTR significantly impaired output levels (Fig. 7B and Supplementary Fig. S7B). When combined with the native YB_TATAp, both the Inactive ${5^{\prime}}$UTR and the RND ${5^{\prime}}$UTR demonstrated similar performance, while removal of the ${5^{\prime}}$UTR abolished output production (Fig. 7C and Supplementary Fig. S7C). When combined with the candidate regulatory elements, the RND ${5^{\prime}}$UTR demonstrated superior performance compared to the Inactive ${5^{\prime}}$UTR, while removal of the ${5^{\prime}}$UTR abolished output production (Fig. 7C and Supplementary Fig. S7C). The miniCMVp and lateADEp are long and contain their own ${5^{\prime}}$UTRs, whereas the TSS of emCMVp, YB_TATAp, and candidate regulatory elements are positioned in very close proximity to the ATG of the output gene, thereby lacking an internal ${5^{\prime}}$UTR sequence. These results are consistent with the established importance of the ${5^{\prime}}$UTR in gene regulation [93–95], and emphasize that the ${5^{\prime}}$UTR is an integral part of minimal promoter engineering, useful to fine-tune gene expression.

### Minimal promoter activity patterns are broadly maintained across cell types

Minimal promoter performance is frequently preserved but can vary across cell types. To further evaluate cell-type dependency, we quantified the activity of selected minimal promoter variants in additional human cell lines representing distinct tissues and cellular states (Fig. 8A–C and Supplementary Fig. S8A–C). For this purpose, we used metastatic breast adenocarcinoma cells (MDA-MB-231), ovarian adenocarcinoma cells (SK-OV-3), non-malignant immortalized lung epithelial cells (BEAS-2B), primary adult human dermal fibroblasts (aHDF), and hTERT-immortalized human pancreatic duct epithelial cells (HPNE). To examine diverse minimal promoter activity patterns and architectures, we tested native, enhanced, and attenuated variants of lateADEp, YB_TATAp, and miniCMVp, as well as the ultrashort TATAv9 candidate regulatory element.

The YB_TATAp(Δ−35 to −32) demonstrated attenuated activity across all cell types (Fig. 8A and Supplementary Fig. S8A), consistent with its performance in HeLa, HaCaT, and HEK293T (Fig. 2C). The YB_TATAp(Δ−23 to −17) demonstrated enhanced activity compared to the native YB_TATAp in most cell types but was comparable to the native YB_TATAp in MDA-MB-231, HaCaT, and HEK293T. Compared to the native YB_TATAp, TATAv9 demonstrated enhanced activity in SK-OV-3, BEAS-2B, aHDF, and HPNE; similar activity in MDA-MB-231, HeLa, and HEK293T; and attenuated activity in HaCaT. The miniCMVp(Δ−52 to −43, −31 to −25, −1 to +57) (Fig. 8B and Supplementary Fig. S8B) demonstrated attenuated activity across all eight cell lines. The emCMVp activity was comparable to that of native miniCMVp across all additional cell types, excluding BEAS-2B cells, in which this variant demonstrated enhanced activity, consistent with its performance in HeLa, HaCaT, and HEK293T (Fig. 3B). Finally, lateADEp(Δ−35 to −32) maintained significantly enhanced activity compared to native lateADEp in most cell lines (Fig. 8C and Supplementary Fig. S8C), consistent with its performance in HeLa, HaCaT, and HEK293T (Fig. 4B). The lateADEp(Δ−17 to −11) demonstrated activity levels comparable to the native lateADEp across most cell lines but was attenuated in HeLa and HEK293T.

Collectively, these data demonstrate that while engineered minimal promoter activity patterns are frequently conserved, their performance can vary between cell types.

### Mapping of transcription start sites of native and engineered variants

Modifications to minimal promoter sequences can alter TSS selection, thereby changing the ${5^{\prime}}$ UTR and potentially affecting post-transcriptional mRNA regulation, including mRNA stability and translation efficiency. Therefore, empirical TSS mapping is essential to determine whether measured transgene levels reflect only alterations in minimal promoter activity or additional regulatory mechanisms. To this end, we used HeLa-derived samples for sequence-resolved ${5^{\prime}}$ RACE and NGS mapping of TSS positions across selected native and engineered minimal promoters encoded upstream of the ${5^{\prime}}$UTR RND [96–99].

As expected, alterations to minimal promoter sequences shifted the dominant TSS coordinates (Fig. 9). Specifically, the dominant TSS of the native miniCMVp was positioned about 60 bp upstream of the ${5^{\prime}}$UTR RND sequence, resulting in an ~52 bp minimal promoter and ~116 bp actual ${5^{\prime}}$UTR that includes the ${5^{\prime}}$UTR RND (~168 bp total). The dominant TSS of the emCMVp was positioned ~3 bp downstream of the ${5^{\prime}}$UTR RND sequence, resulting in an ~40 bp minimal promoter and ~57 bp actual ${5^{\prime}}$UTR that includes the ${5^{\prime}}$UTR RND (~99 bp total). The dominant TSS of the native YB_TATAp was positioned ~15 bp downstream of the ${5^{\prime}}$UTR RND sequence, resulting in an ~39 bp minimal promoter and ~44 bp actual ${5^{\prime}}$UTR that includes the ${5^{\prime}}$UTR RND (~84 bp total). The dominant TSS of the YB_TATAp(SMAD2→ΔTCTAGA,Δ−18 to −15) was positioned ~21 bp downstream of the ${5^{\prime}}$UTR RND sequence, resulting in an ~43 bp minimal promoter and ~39 bp actual ${5^{\prime}}$UTR that includes the ${5^{\prime}}$UTR RND (~82 bp total). The dominant TSS of the TATAv9 was positioned ~23 bp downstream of the ${5^{\prime}}$UTR RND sequence, resulting in an ~31 bp minimal promoter and ~36 bp actual ${5^{\prime}}$UTR that includes the ${5^{\prime}}$UTR RND (~67 bp total). The dominant TSS of the native lateADEp was positioned ~5 bp upstream of the ${5^{\prime}}$UTR RND sequence, resulting in an ~46 bp minimal promoter and ~64 bp actual ${5^{\prime}}$UTR that includes the ${5^{\prime}}$UTR RND (~110 bp total). The dominant TSS of the lateADEp(Δ−35 to −32) was positioned ~24 bp downstream of the ${5^{\prime}}$UTR RND sequence, resulting in an ~73 bp minimal promoter and an ~36 bp actual ${5^{\prime}}$UTR that includes the ${5^{\prime}}$UTR RND (~109 bp total).

**Figure 9.**
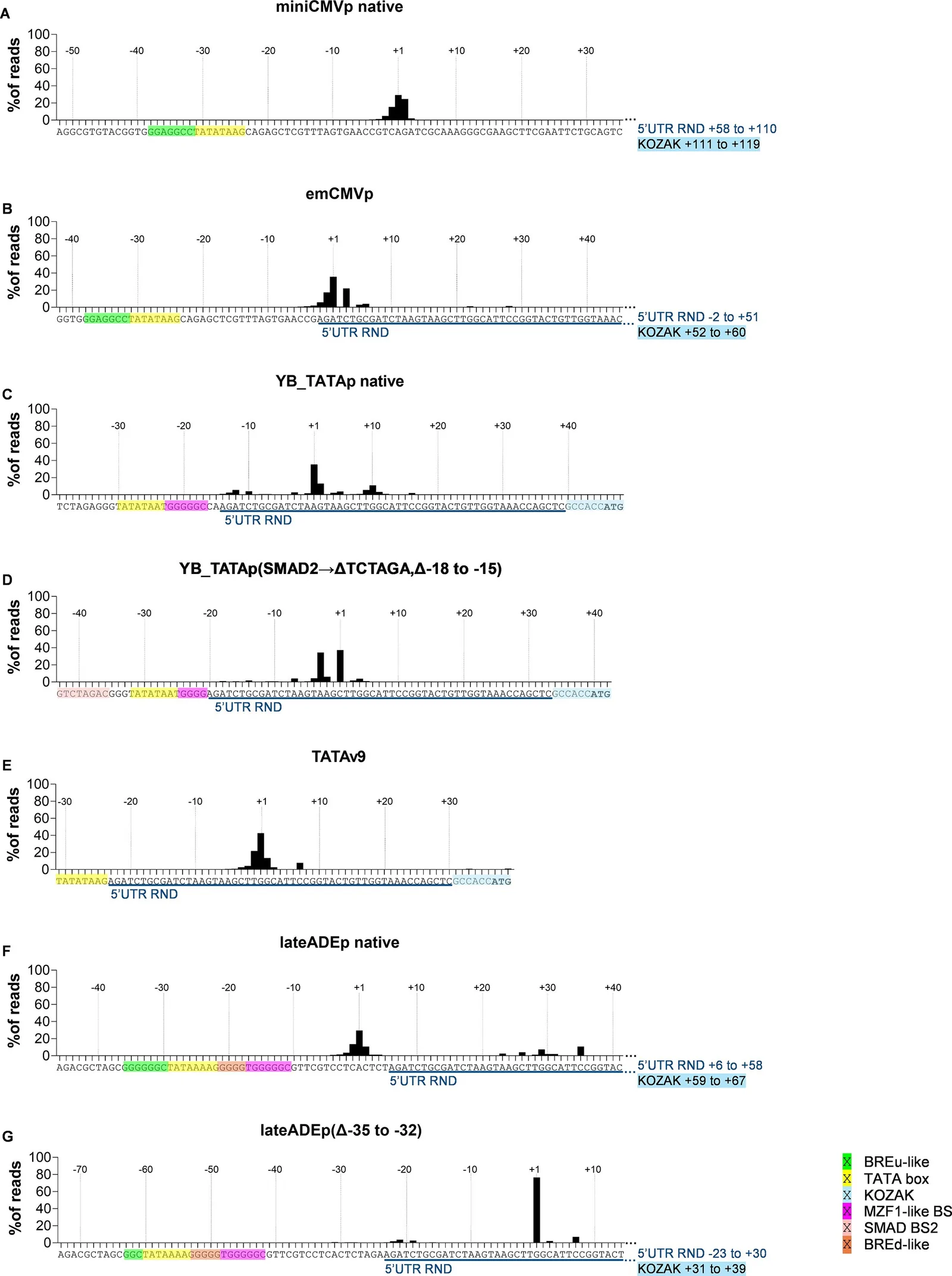
Mapping the TSSs of native and engineered minimal promoter variants. A ${5^{\prime}}$RACE-seq was used to map the TSSs of native and engineered minimal promoter variants in HeLa cells. (**A**) miniCMVp native, (**B**) emCMVp, (**C**) YB_TATAp native, (**D**) YB_TATAp(SMAD2→ΔTCTAGA, Δ−18 to −15), (**E**) TATAv9, (**F**) lateADEp native, and (**G**) lateADEp(Δ−35 to −32). For all panels, histograms show the percentage of mapped reads plotted relative to the main TSS, assigned as +1. The corresponding minimal promoter sequence is displayed beneath each histogram; the RND ${5^{\prime}}$UTR and KOZAK sequences are annotated within each panel, and their positions relative to +1 appear next to the panel when located outside the displayed sequence. Full sequences are provided in Tables 1, 4, and 7. The main TSS position of each minimal promoter was assigned to the ${5^{\prime}}$ nucleotide of the read with the highest count after adapter and poly-T trimming, as described in the “Materials and methods” section.

These data indicate that TSS selection is promoter-dependent, thereby defining the actual ${5^{\prime}}$ UTR sequence in each variant. The dominant TSS for most minimal promoter variants was located ~30 bp downstream of the ${5^{\prime}}$T of the TATA box. The TATAv9 candidate regulatory element also displayed a similar TATA-to-TSS spacing, placing its TSS within the ${5^{\prime}}$ UTR, as observed for the native YB_TATAp. However, the dominant TSS of lateADEp(Δ−35 to −32) was located ~60 bp downstream of the ${5^{\prime}}$ T of the TATA box, suggesting that the retained canonical TATA box may not determine the dominant TSS in this variant. In this case, a TCTAGAAG sequence located ~30 bp upstream of the dominant TSS may function as a non-canonical TATA-like motif. However, this hypothesis requires experimental validation using an appropriate functional assay.

Together, these findings indicate that minimal promoter modifications can shift the dominant TSS, thereby changing the length and sequence composition of the transcribed ${5^{\prime}}$ UTR. Thus, differences in reporter protein levels among minimal promoter variants may also reflect changes in post-transcriptional mRNA regulation. Accordingly, only limited conclusions regarding the functional contribution of specific sequence elements can be drawn from reporter fluorescence alone, particularly for variants in which the TSS was not experimentally mapped. Finally, since minimal promoter elements and ${5^{\prime}}$ UTR sequences function as an integrated regulatory unit, predictable tuning of transgene levels would require predefined regulatory units comprising specific minimal promoters and ${5^{\prime}}$ UTRs.

## Discussion

In this study, we established a systematic engineering framework that provides tunable, enhanced, and candidate regulatory elements for optimized transgene expression in mammalian cells. As a basis for this approach, we used lentiviral vectors that sequentially encode the synthetic AHR:ARNT enhancer, a minimal promoter variant, and an RND ${5^{\prime}}$ UTR, which together regulate expression of the mKate2 gene. We validated that mKate2 fluorescence measured by flow cytometry reflects the mKate2 RNA levels (Supplementary Fig. S9A and B) and that relative mKate2 fluorescence levels in this experimental setup are not affected by vector copy number (Supplementary Fig. S9A and B). We found that in all cell lines, promoter activity was higher in the G1 phase than in the S or G2 phases (Supplementary Fig. S9C). Yet, this cell-phase dependency was also observed for the stable hUbCp housekeeping promoter, which comprises a native genomic segment of the human ubiquitin C promoter. This pattern suggests that the observed cell-phase dependency reflects a broader cellular phenomenon rather than a unique property of the engineered promoters, consistent with known global regulation of gene expression during the cell cycle [100]. Together, these results further support the use of mKate2 fluorescence levels as a reliable indicator of minimal promoter performance.

We found that TATA box substitution enables the modulation of gene expression, although these motifs are not universally interchangeable. Sequences conserved among minimal promoters and canonical regulatory motifs may function as modular components that support tunable context-dependent activity. Notably, shorter minimal promoters with enhanced activity can be generated by deletion of redundant sequences. We further demonstrated the feasibility of 7–8 bp candidate regulatory elements, which enable potent and tunable gene expression when paired with readily available ${5^{\prime}}$UTRs. These findings highlight a modular architecture of minimal promoters, in which the composition and sequence of regulatory motifs are interchangeable, enabling tunable transcriptional activity.

Characterizing the mechanisms that determine the function of minimal promoter variants would improve predictability of engineering outcomes and improve our understanding of transcriptional regulation. However, several limitations warrant consideration. Deleting putative regulatory elements may inadvertently create new sequence contexts or novel combinations of core promoter elements and change minimal promoter length, complicating interpretation of the observed effects. Using substitution mutations rather than deletions could minimize this concern. However, ${5^{\prime}}$ RACE analysis indicates that any modification to the minimal promoter sequence can shift the dominant TSS, thereby altering both the length and sequence composition of the transcribed ${5^{\prime}}$ UTR. Consequently, differences in fluorescent reporter protein levels may also reflect changes in TSS selection that affect post-transcriptional mRNA regulation rather than solely the intrinsic activity of a putative deleted or added sequence motif. We addressed changes in translation efficiency by quantifying mRNA levels generated by each minimal promoter variant, in addition to measuring protein concentrations (Supplementary Fig. S9A and B). Nevertheless, only limited conclusions can be drawn from reporter fluorescence alone, underscoring the need for TSS mapping, mutation analysis, and functional assays to determine the function of specific sequence elements. Moreover, we performed TSS mapping solely in HeLa cells. Therefore, additional studies are needed to determine whether TSS selection is fully conserved across cell types and cellular states.

In summary, this study demonstrates that the modular structure of minimal promoters and candidate regulatory elements enables fine-tuning of gene expression. Moreover, minimal promoter functionality is often cell-type dependent and could be implemented to improve the tissue specificity of synthetic promoters. These findings establish a rational minimal promoter engineering framework that would support programmable gene expression, essential for applications in fundamental biological research, biotechnology, and biomedicine.

## Extended data

### Extended data Table 1: Broad analysis of all minimal promoters with ElemeNT2023

Depicted are the analyses of minimal promoters (Tables 3 and 4 ) together with all surrounding sequences (Enhancer region, RND ${5^{\prime}}$UTR, KOZAK, and restriction enzyme sites) to identify minimal promoter elements in said sequences, done using Juven-Gershon’s ElemeNT 2023 tool.

### Extended data Fig. 1: TSS mapping analysis

Depicted are the results and analysis of the NGS, the results of which are presented in Fig. 9.

### Extended data Fig. 2: Broad analysis of all minimal promoters with ElemeNT2023

Depicted are the analyses of the minimal promoters and ${5^{\prime}}$UTR combinations, the results of which are presented in Fig. 7. Analysis was performed using Juven-Gershon’s ElemeNT 2023 tool.

### Extended data Fig. 3: Effect of insert orientation on minimal promoter activity.

(A) Schematic representation of lentiviral constructs containing the enhancer-minimal promoter-${5^{\prime}}$UTR-mKate2 cassette in either the forward (top) or reverse (bottom) orientation relative to the CMVp-driven viral backbone.

(B) Weighted median mKate2 expression levels of YB_TATAp and lateADEp constructs were compared between forward and reverse orientations. No significant differences were detected (ns, two-tailed *t*-test).

Panel (A) was created in BioRender. Nissim, L. (2026) https://BioRender.com/sscn9yw.

## Supplementary Material

gkag1052_Supplemental_Files

## Data Availability

Lead contact: Requests for further information, resources, and reagents should be directed to and will be fulfilled by the lead contact, Lior Nissim (lior.nissim@mail.huji.ac.il). Materials availability: Full sequences for all plasmids used in this study are detailed in GenBank format in the supplementary ZIP file “PlasmidSequences.zip.” Plasmids and plasmid sequences were also deposited to Addgene and can be found at: https://www.addgene.org/browse/article/28275426/. Data and code availability The computational code generated during this study and raw sequencing data (NGS) generated in this study were deposited in the Gene Expression Omnibus (GEO) database under accession code GSE335462 and can be accessed in this link: https://www.ncbi.nlm.nih.gov/geo/query/acc.cgi?acc=GSE335462 Any additional data supporting the findings of this study are available within the paper and its Supplementary information, or can be provided by the lead contact upon request.
