# Supplementary material for: Systematic engineering of synthetic minimal promoters for tunable transgene expression in mammalian cells
Source: Nucleic Acids Res. 2026 Nov 4;54(20):gkag1052. doi: 10.1093/nar/gkag1052 (PMC13732139; doi:10.1093/nar/gkag1052)
Supplement: Supplementary file 1 [file gkag1052_supplemental_files.zip › Extended Data Figure 2 Revision 2.pdf]

>lateADEp-RND_5UTR

GGCGCGCCAGACGCTAGCGGGGGGCTATAAAAGGGGGTGGGGGCGTTCGTCCTCACTCTAGATCTGCGATCTAAGTAAGCTTGGCATTCCGGTACTGTTGGTAAACCAGCTCGCCACCATG

A possible combination, input sequence position 26 to 60:

TATAAAAGGGGGTGGGGGCGTTCGTCCTCACTCT

A possible combination, input sequence position 7 to 42:

CCAGACGCTAGCGGGGGGCTATAAAAGGGGGTGGG

A possible combination, input sequence position 26 to 65:

TATAAAAGGGGGTGGGGGCGTTCGTCCTCACTCTAGATC

Elements found:					
**Element**	**Start position**	**Sequence**	**PWM score**	**Consensus Match**	
TATA box	26	TATAAAAG	10.86	8 out of 8	distance to Mammalian Initiator A+1 (position 53) is 30<br>distance to Mammalian Initiator A+1 (position 58) is 35
BRE upstream	2	GCGCGCC	12.37	7 out of 7	
BRE upstream	109	GCTCGCC	6.18	6 out of 7	
BRE downstream	37	GTGGGGG	4.95	7 out of 7	
BRE downstream	33	GGGGGTG	4.25	6 out of 7	
Mammalian Initiator	53	TCACTCT	6.19	7 out of 7	
Mammalian Initiator	106	CCAGCTC	5.82	6 out of 7	
Mammalian Initiator	7	CCAGACG	4.82	6 out of 7	
DPE	36	GGTGGG	1.54	4 out of 6	
Mammalian Initiator	58	CTAGATC	4.21	7 out of 7	
Mammalian Initiator	84	GCATTCC	4.07	6 out of 7	
Mammalian Initiator	89	CCGGTAC	3.11	5 out of 7	
Mammalian Initiator	114	CCACCAT	2.62	5 out of 7	
Mammalian Initiator	47	TCGTCCT	0.71	5 out of 7	

>lateADEp-none
GGAGACGCTAGCGGGGGGCTATAAAAGGGGGTGGGGGCGTTCGTCCTCACTCTAGAGCCACCATG

A possible combination, input sequence position 20 to 54:
TATAAAAGGGGGTGGGGGCGTTCGTCCTCACTCT

A possible combination, input sequence position 20 to 59:
TATAAAAGGGGGTGGGGGCGTTCGTCCTCACTCTAGAGC

A possible combination, input sequence position 20 to 65:
TATAAAAGGGGGTGGGGGCGTTCGTCCTCACTCTAGAGCCACCAT

Elements found:					
**Element**	**Start position**	**Sequence**	**PWM score**	**Consensus Match**	
TATA box	20	TATAAAAG	10.86	8 out of 8	distance to Mammalian Initiator A+1 (position 47) is 30<br>distance to Mammalian Initiator A+1 (position 52) is 35<br>distance to Mammalian Initiator A+1 (position 58) is 41
BRE downstream	31	GTGGGGG	4.95	7 out of 7	
BRE downstream	27	GGGGGTG	4.25	6 out of 7	
Mammalian Initiator	47	TCACTCT	6.19	7 out of 7	
Mammalian Initiator	52	CTAGAGC	2.77	6 out of 7	
Mammalian Initiator	58	CCACCAT	2.62	5 out of 7	
Mammalian Initiator	41	TCGTCCT	0.71	5 out of 7	

>lateADEp-non-active

GGAGACGCTAGCGGGGGGCTATAAAAGGGGGTGGGGGCGTTCGTCCTCACTCTAGAAATTGGTGCCGCGAAGAAGCCTGGTTAAGGAGAACTCTCGGCGTAAGCCACCATG

A possible combination, input sequence position 20 to 54:

TATAAAAGGGGGTGGGGGCGTTCGTCCTCACTCT

Elements found:					
**Element**	**Start position**	**Sequence**	**PWM score**	**Consensus Match**	
TATA box	20	TATAAAAG	10.86	8 out of 8	distance to Mammalian Initiator A+1 (position 47) is 30
BRE downstream	31	GTGGGGG	4.95	7 out of 7	
BRE downstream	27	GGGGGTG	4.25	6 out of 7	
Mammalian Initiator	47	TCACTCT	6.19	7 out of 7	
Mammalian Initiator	104	CCACCAT	2.62	5 out of 7	
Mammalian Initiator	41	TCGTCCT	0.71	5 out of 7	

>LateADEp_del_4-RND_5UTR

GGAGACGCTAGCGGCTATAAAAGGGGGTGGGGGCGTTCGTCCTCACTCTAGAAGATCTGCGATCTAAGTAAGCTTGGCATTCCGGTACTGTTGGTAAACCAGCTCGCCACCATG

A possible combination, input sequence position 16 to 78:

TATAAAAGGGGGTGGGGGCGTTCGTCCTCACTCTAGAAGATCTGCGATCTAAGTAAGCTTGG

Elements found:					
**Element**	**Start position**	**Sequence**	**PWM score**	**Consensus Match**	
TATA box	16	TATAAAAG	10.86	8 out of 8	distance to Mammalian Initiator A+1 (position 43) is 30
BRE upstream	102	GCTCGCC	6.18	6 out of 7	
BRE downstream	27	GTGGGGG	4.95	7 out of 7	
BRE downstream	23	GGGGGTG	4.25	6 out of 7	
Mammalian Initiator	43	TCACTCT	6.19	7 out of 7	
DPE	72	GCTTGG	3.48	5 out of 6	
Mammalian Initiator	99	CCAGCTC	5.82	6 out of 7	
Mammalian Initiator	77	GCATTCC	4.07	6 out of 7	
Mammalian Initiator	82	CCGGTAC	3.11	5 out of 7	
Mammalian Initiator	107	CCACCAT	2.62	5 out of 7	
Mammalian Initiator	37	TCGTCCT	0.71	5 out of 7	

>LateADEp_del_4-none
GGAGACGCTAGCGGCTATAAAAGGGGGTGGGGGCGTTCGTCCTCACTCTAGAGCCACCATG

A possible combination, input sequence position 16 to 50:
TATAAAAGGGGGTGGGGGCGTTCGTCCTCACTCT

A possible combination, input sequence position 16 to 55:
TATAAAAGGGGGTGGGGGCGTTCGTCCTCACTCTAGAGC

A possible combination, input sequence position 16 to 61:
TATAAAAGGGGGTGGGGGCGTTCGTCCTCACTCTAGAGCCACCAT

Elements found:					
**Element**	**Start position**	**Sequence**	**PWM score**	**Consensus Match**	
TATA box	16	TATAAAAG	10.86	8 out of 8	distance to Mammalian Initiator A+1 (position 43) is 30<br>distance to Mammalian Initiator A+1 (position 48) is 35<br>distance to Mammalian Initiator A+1 (position 54) is 41
BRE downstream	27	GTGGGGG	4.95	7 out of 7	
BRE downstream	23	GGGGGTG	4.25	6 out of 7	
Mammalian Initiator	43	TCACTCT	6.19	7 out of 7	
Mammalian Initiator	48	CTAGAGC	2.77	6 out of 7	
Mammalian Initiator	54	CCACCAT	2.62	5 out of 7	
Mammalian Initiator	37	TCGTCCT	0.71	5 out of 7	

>LateADEp_del_4-non-active
GGAGACGCTAGCGGCTATAAAAGGGGGTGGGGGCGTTCGTCCTCACTCTAGAAATTGGTGCCGCGAAGAAGCCTGGTTAAGGAGAACTCTCGGCGTAAGCCACCATG

A possible combination, input sequence position 16 to 50:
TATAAAAGGGGGTGGGGGCGTTCGTCCTCACTCT

Elements found:					
**Element**	**Start position**	**Sequence**	**PWM score**	**Consensus Match**	
TATA box	16	TATAAAAG	10.86	8 out of 8	distance to Mammalian Initiator A+1 (position 43) is 30
BRE downstream	27	GTGGGGG	4.95	7 out of 7	
BRE downstream	23	GGGGGTG	4.25	6 out of 7	
Mammalian Initiator	43	TCACTCT	6.19	7 out of 7	
Mammalian Initiator	100	CCACCAT	2.62	5 out of 7	
Mammalian Initiator	37	TCGTCCT	0.71	5 out of 7	

>LateADEp_del_7-RND_5UTR
GGAGACGCTAGCCTATAAAAGGGGGTGGGGGCGTTCGTCCTCACTCTAGAAGATCTGCGATCTAAGTAAGCTTGGCATTCCGGTACTGTTGGTAAACCAGCTCGCCACCATG

A possible combination, input sequence position 14 to 76:
TATAAAAGGGGGTGGGGGCGTTCGTCCTCACTCTAGAAGATCTGCGATCTAAGTAAGCTTGG

Elements found:					
**Element**	**Start position**	**Sequence**	**PWM score**	**Consensus Match**	
TATA box	14	TATAAAAG	10.86	8 out of 8	distance to Mammalian Initiator A+1 (position 41) is 30
BRE upstream	100	GCTCGCC	6.18	6 out of 7	
BRE downstream	25	GTGGGGG	4.95	7 out of 7	
BRE downstream	21	GGGGGTG	4.25	6 out of 7	
Mammalian Initiator	41	TCACTCT	6.19	7 out of 7	
DPE	70	GCTTGG	3.48	5 out of 6	
Mammalian Initiator	97	CCAGCTC	5.82	6 out of 7	
Mammalian Initiator	75	GCATTCC	4.07	6 out of 7	
Mammalian Initiator	80	CCGGTAC	3.11	5 out of 7	
Mammalian Initiator	8	CTAGCCT	2.72	6 out of 7	
Mammalian Initiator	105	CCACCAT	2.62	5 out of 7	
Mammalian Initiator	35	TCGTCCT	0.71	5 out of 7	

>LateADEp_del_7-none
GGAGACGCTAGCCTATAAAAGGGGGTGGGGGCGTTCGTCCTCACTCTAGAGCCACCATG

A possible combination, input sequence position 14 to 48:
TATAAAAGGGGGTGGGGGCGTTCGTCCTCACTCT

A possible combination, input sequence position 14 to 53:
TATAAAAGGGGGTGGGGGCGTTCGTCCTCACTCTAGAGC

A possible combination, input sequence position 14 to 59:
TATAAAAGGGGGTGGGGGCGTTCGTCCTCACTCTAGAGCCACCAT

Elements found:					
**Element**	**Start position**	**Sequence**	**PWM score**	**Consensus Match**	
TATA box	14	TATAAAAG	10.86	8 out of 8	distance to Mammalian Initiator A+1 (position 41) is 30<br>distance to Mammalian Initiator A+1 (position 46) is 35<br>distance to Mammalian Initiator A+1 (position 52) is 41
BRE downstream	25	GTGGGGG	4.95	7 out of 7	
BRE downstream	21	GGGGGTG	4.25	6 out of 7	
Mammalian Initiator	41	TCACTCT	6.19	7 out of 7	
Mammalian Initiator	46	CTAGAGC	2.77	6 out of 7	
Mammalian Initiator	8	CTAGCCT	2.72	6 out of 7	
Mammalian Initiator	52	CCACCAT	2.62	5 out of 7	
Mammalian Initiator	35	TCGTCCT	0.71	5 out of 7	

>LateADEp_del_7-non-active
GGAGACGCTAGCCTATAAAAGGGGGTGGGGGCGTTCGTCCTCACTCTAGAAATTGGTGCCGCGAAGAAGCCTGGTTAAGGAGAACTCTCGGCGTAAGCCACCATG

A possible combination, input sequence position 14 to 48:
TATAAAAGGGGGTGGGGGCGTTCGTCCTCACTCT

Elements found:					
**Element**	**Start position**	**Sequence**	**PWM score**	**Consensus Match**	
TATA box	14	TATAAAAG	10.86	8 out of 8	distance to Mammalian Initiator A+1 (position 41) is 30
BRE downstream	25	GTGGGGG	4.95	7 out of 7	
BRE downstream	21	GGGGGTG	4.25	6 out of 7	
Mammalian Initiator	41	TCACTCT	6.19	7 out of 7	
Mammalian Initiator	8	CTAGCCT	2.72	6 out of 7	
Mammalian Initiator	98	CCACCAT	2.62	5 out of 7	
Mammalian Initiator	35	TCGTCCT	0.71	5 out of 7	

>miniCMVp-RND_5UTR
GGGGCGCGCCAGGCGTGTACGGTGGGAGGCCTATATAAGCAGAGCTCGTTTAGTGAACCGTCAGATCGCAAAGGGCGAAGCTTCGAATTCTGCAGTCGACGGTACCGCGGGCCACCGGTAGATCTGCGATCTAAGTAAGCTT
GGCATTCCGGTACTGTTGGTAAACCAGCTCGCCACCATG

A possible combination, input sequence position 25 to 68:
GGAGGCCTATATAAGCAGAGCTCGTTTAGTGAACCGTCAGATC

A possible combination, input sequence position 83 to 118:
TCGAATTCTGCAGTCGACGGTACCGCGGGCCACCG

A possible combination, input sequence position 25 to 40:
GGAGGCCTATATAAG

Elements found:					
**Element**	**Start position**	**Sequence**	**PWM score**	**Consensus Match**	
TATA box	32	TATATAAG	10.86	8 out of 8	distance to Mammalian Initiator A+1 (position 61) is 32<br>distance to BRE upstream (position 25) is 0
BRE upstream	4	GCGCGCC	12.37	7 out of 7	
BRE upstream	107	GCGGGCC	9.59	6 out of 7	
BRE upstream	2	GGGCGCG	7.53	6 out of 7	
BRE upstream	25	GGAGGCC	7.09	6 out of 7	
BRE upstream	169	GCTCGCC	6.18	6 out of 7	
Mammalian Initiator	166	CCAGCTC	5.82	6 out of 7	
Mammalian Initiator	61	TCAGATC	5.70	7 out of 7	
Mammalian Initiator	144	GCATTCC	4.07	6 out of 7	
Mammalian Initiator	9	CCAGGCG	4.06	5 out of 7	
Mammalian Initiator	84	CGAATTC	3.14	6 out of 7	
Mammalian Initiator	149	CCGGTAC	3.11	5 out of 7	
Mammalian Initiator	92	GCAGTCG	2.64	5 out of 7	
Mammalian Initiator	174	CCACCAT	2.62	5 out of 7	

Mammalian Initiator	112	CCACCGG	2.15	4 out of 7	
Mammalian Initiator	39	GCAGAGC	1.82	5 out of 7	
Mammalian Initiator	83	TCGAATT	1.02	6 out of 7	
DPE	112	CCACCG	0.88	4 out of 6	
Mammalian Initiator	118	GTAGATC	0.89	6 out of 7	
Mammalian Initiator	115	CCGGTAG	0.79	4 out of 7	

>miniCMVp-none
GGAGGCGTGTACGGTGGGAGGCCTATATAAGCAGAGCTCGTTTAGTGAACCGTCAGATCGCAAAGGGCGAAGCTTCGAATTCTGCAGTCGACGGTACCGCGGGCCGCCACCATG

A possible combination, input sequence position 17 to 60:
GGAGGCCTATATAAGCAGAGCTCGTTTAGTGAACCGTCAGATC

A possible combination, input sequence position 17 to 32:
GGAGGCCTATATAAG

Elements found:					
**Element**	**Start position**	**Sequence**	**PWM score**	**Consensus Match**	
TATA box	24	TATATAAG	10.86	8 out of 8	distance to Mammalian Initiator A+1 (position 53) is 32<br>distance to BRE upstream (position 17) is 0
BRE upstream	102	GGCCGCC	9.87	6 out of 7	
BRE upstream	99	GCGGGCC	9.59	6 out of 7	
BRE upstream	17	GGAGGCC	7.09	6 out of 7	
Mammalian Initiator	53	TCAGATC	5.70	7 out of 7	
Mammalian Initiator	76	CGAATTC	3.14	6 out of 7	
Mammalian Initiator	84	GCAGTCG	2.64	5 out of 7	
Mammalian Initiator	107	CCACCAT	2.62	5 out of 7	
Mammalian Initiator	31	GCAGAGC	1.82	5 out of 7	
Mammalian Initiator	104	CCGCCAC	1.30	4 out of 7	
Mammalian Initiator	75	TCGAATT	1.02	6 out of 7	

>miniCMVp-non-active
GGAGGCGTGTACGGTGGGAGGCCTATATAAGCAGAGCTCGTTTAGTGAACCGTCAGATCGCAAAGGGCGAAGCTTCGAATTCTGCAGTCGACGGTACCGCGGGCCAATTGGTGCCGCGAAGAAGCCTGGTTAAGGAGAACTC
TCGGCGTAAGCCACCATG

A possible combination, input sequence position 17 to 60:
GGAGGCCTATATAAGCAGAGCTCGTTTAGTGAACCGTCAGATC

A possible combination, input sequence position 17 to 32:
GGAGGCCTATATAAG

Elements found:					
**Element**	**Start position**	**Sequence**	**PWM score**	**Consensus Match**	
TATA box	24	TATATAAG	10.86	8 out of 8	distance to Mammalian Initiator A+1 (position 53) is 32<br>distance to BRE upstream (position 17) is 0
BRE upstream	99	GCGGGCC	9.59	6 out of 7	
BRE upstream	17	GGAGGCC	7.09	6 out of 7	
Mammalian Initiator	53	TCAGATC	5.70	7 out of 7	
Mammalian Initiator	104	CCAATTG	4.63	6 out of 7	
Mammalian Initiator	76	CGAATTC	3.14	6 out of 7	
Mammalian Initiator	84	GCAGTCG	2.64	5 out of 7	
Mammalian Initiator	153	CCACCAT	2.62	5 out of 7	
Mammalian Initiator	31	GCAGAGC	1.82	5 out of 7	
Mammalian Initiator	75	TCGAATT	1.02	6 out of 7	

>emCMVp-RND_5UTR
GGGGTGGGAGGCCTATATAAGCAGAGCTCGTTTAGTGAACCGAGATCTGCGATCTAAGTAAGCTTGGCATTCCGGTACTGTTGGTAAACCAGCTCGCCACCATG

A possible combination, input sequence position 7 to 48:
GGAGGCCTATATAAGCAGAGCTCGTTTAGTGAACCGAGATC

A possible combination, input sequence position 21 to 56:
GCAGAGCTCGTTTAGTGAACCGAGATCTGCGATCT

A possible combination, input sequence position 7 to 22:
GGAGGCCTATATAAG

Elements found:					
**Element**	**Start position**	**Sequence**	**PWM score**	**Consensus Match**	
TATA box	14	TATATAAG	10.86	8 out of 8	distance to Mammalian Initiator A+1 (position 41) is 30<br>distance to BRE upstream (position 7) is 0
BRE upstream	7	GGAGGCC	7.09	6 out of 7	
BRE upstream	92	GCTCGCC	6.18	6 out of 7	
Mammalian Initiator	89	CCAGCTC	5.82	6 out of 7	
Mammalian Initiator	67	GCATTCC	4.07	6 out of 7	
Mammalian Initiator	72	CCGGTAC	3.11	5 out of 7	
Mammalian Initiator	41	CGAGATC	2.77	6 out of 7	
Mammalian Initiator	97	CCACCAT	2.62	5 out of 7	
Mammalian Initiator	21	GCAGAGC	1.82	5 out of 7	
DPE	50	CGATCT	3.12	5 out of 6	

>emCMVp-none
GGGGTGGGAGGCCTATATAAGCAGAGCTCGTTTAGTGAACCGGCCACCATG

A possible combination, input sequence position 7 to 51:
GGAGGCCTATATAAGCAGAGCTCGTTTAGTGAACCGGCCACCAT

A possible combination, input sequence position 7 to 47:
GGAGGCCTATATAAGCAGAGCTCGTTTAGTGAACCGGCCA

A possible combination, input sequence position 7 to 22:
GGAGGCCTATATAAG

Elements found:					
**Element**	**Start position**	**Sequence**	**PWM score**	**Consensus Match**	
TATA box	14	TATATAAG	10.86	8 out of 8	distance to Mammalian Initiator A+1 (position 44) is 33<br>distance to Mammalian Initiator A+1 (position 40) is 29<br>distance to BRE upstream (position 7) is 0
BRE upstream	7	GGAGGCC	7.09	6 out of 7	
Mammalian Initiator	44	CCACCAT	2.62	5 out of 7	
Mammalian Initiator	21	GCAGAGC	1.82	5 out of 7	
Mammalian Initiator	40	CCGGCCA	0.89	4 out of 7	

>emCMVp-non-active
GGGGTGGGAGGCCTATATAAGCAGAGCTCGTTTAGTGAACCGAATTGGTGCCGCGAAGAAGCCTGGTTAAGGAGAACTCTCGGCGTAAGCCACCATG

A possible combination, input sequence position 7 to 47:
GGAGGCCTATATAAGCAGAGCTCGTTTAGTGAACCGAATT

A possible combination, input sequence position 7 to 48:
GGAGGCCTATATAAGCAGAGCTCGTTTAGTGAACCGAATTG

A possible combination, input sequence position 7 to 22:
GGAGGCCTATATAAG

Elements found:					
**Element**	**Start position**	**Sequence**	**PWM score**	**Consensus Match**	
TATA box	14	TATATAAG	10.86	8 out of 8	distance to Mammalian Initiator A+1 (position 40) is 29<br>distance to Mammalian Initiator A+1 (position 41) is 30<br>distance to BRE upstream (position 7) is 0
BRE upstream	7	GGAGGCC	7.09	6 out of 7	
Mammalian Initiator	90	CCACCAT	2.62	5 out of 7	
Mammalian Initiator	40	CCGAATT	1.91	6 out of 7	
Mammalian Initiator	21	GCAGAGC	1.82	5 out of 7	
Mammalian Initiator	41	CGAATTG	0.82	5 out of 7	

>YB_TATA-RND_5UTR

GGCGCGCCTCTAGAGGGTATATAATGGGGGCCAAGATCTGCGATCTAAGTAAGCTTGGCATTCCGGTACTGTTGGTAAACCAGCTCGCCACCATG

No combinations of core promoter elements were detected in the input sequence.

Elements found:				
**Element**	**Start position**	**Sequence**	**PWM score**	**Consensus Match**
TATA box	18	TATATAAT	8.30	7 out of 8
BRE upstream	2	GCGCGCC	12.37	7 out of 7
BRE upstream	26	GGGGGCC	8.62	6 out of 7
BRE upstream	83	GCTCGCC	6.18	6 out of 7
Mammalian Initiator	80	CCAGCTC	5.82	6 out of 7
Mammalian Initiator	58	GCATTCC	4.07	6 out of 7
Mammalian Initiator	63	CCGGTAC	3.11	5 out of 7
Mammalian Initiator	88	CCACCAT	2.62	5 out of 7
Mammalian Initiator	32	CAAGATC	2.21	6 out of 7
Mammalian Initiator	31	CCAAGAT	1.75	5 out of 7

>YB_TATA-none

GGTCTAGAGGGTATATAATGGGGGCCAGCCACCATG

No combinations of core promoter elements were detected in the input sequence.

Elements found:				
**Element**	**Start position**	**Sequence**	**PWM score**	**Consensus Match**
TATA box	12	TATATAAT	8.30	7 out of 8
BRE upstream	20	GGGGGCC	8.62	6 out of 7
BRE upstream	24	GCCAGCC	8.44	5 out of 7
Mammalian Initiator	25	CCAGCCA	3.50	5 out of 7
Mammalian Initiator	29	CCACCAT	2.62	5 out of 7

>YB_TATA-non-active

GGTCTAGAGGGTATATAATGGGGGCCAAATTGGTGCCGCGAAGAAGCCTGGTTAAGGAGAACTCTCGGCGTAAGCCACCATG

No combinations of core promoter elements were detected in the input sequence.

Elements found:				
**Element**	**Start position**	**Sequence**	**PWM score**	**Consensus Match**
TATA box	12	TATATAAT	8.30	7 out of 8
BRE upstream	20	GGGGGCC	8.62	6 out of 7
Mammalian Initiator	25	CCAAATT	4.52	7 out of 7
Mammalian Initiator	75	CCACCAT	2.62	5 out of 7

>TATA_BOX_var_1-RND_5UTR
GGTATAAAAAGATCTGCGATCTAAGTAAGCTTGGCATTCCGGTACTGTTGGTAAACCAGCTCGCCACCATG

A possible combination, input sequence position 3 to 41:
TATAAAAAGATCTGCGATCTAAGTAAGCTTGGCATTCC

A possible combination, input sequence position 3 to 46:
TATAAAAAGATCTGCGATCTAAGTAAGCTTGGCATTCCGGTAC

Elements found:					
**Element**	**Start position**	**Sequence**	**PWM score**	**Consensus Match**	
TATA box	3	TATAAAAA	10.30	8 out of 8	distance to Mammalian Initiator A+1 (position 34) is 34<br>distance to Mammalian Initiator A+1 (position 39) is 39
BRE upstream	59	GCTCGCC	6.18	6 out of 7	
Mammalian Initiator	56	CCAGCTC	5.82	6 out of 7	
Mammalian Initiator	34	GCATTCC	4.07	6 out of 7	
Mammalian Initiator	39	CCGGTAC	3.11	5 out of 7	
Mammalian Initiator	64	CCACCAT	2.62	5 out of 7	

>TATA_BOX_var_1-none

GGTATAAAAGCCACCATG

No combinations of core promoter elements were detected in the input sequence.

Elements found:				
**Element**	**Start position**	**Sequence**	**PWM score**	**Consensus Match**
TATA box	3	TATAAAAG	10.86	8 out of 8
Mammalian Initiator	11	CCACCAT	2.62	5 out of 7

>TATA_BOX_var_1-non-active

GGTATAAAAAATTGGTGCCGCGAAGAAGCCTGGTTAAGGAGAACTCTCGGCGTAAGCCACCATG

No combinations of core promoter elements were detected in the input sequence.

Elements found:				
**Element**	**Start position**	**Sequence**	**PWM score**	**Consensus Match**
TATA box	3	TATAAAAA	10.30	8 out of 8
Mammalian Initiator	57	CCACCAT	2.62	5 out of 7

>TATA_BOX_var_2-RND_5UTR
GGTATAAATAGATCTGCGATCTAAGTAAGCTTGGCATTCCGGTACTGTTGGTAAACCAGCTCGCCACCATG

A possible combination, input sequence position 3 to 41:
TATAAATAGATCTGCGATCTAAGTAAGCTTGGCATTCC

A possible combination, input sequence position 3 to 46:
TATAAATAGATCTGCGATCTAAGTAAGCTTGGCATTCCGGTAC

Elements found:					
**Element**	**Start position**	**Sequence**	**PWM score**	**Consensus Match**	
TATA box	3	TATAAATA	7.49	7 out of 8	distance to Mammalian Initiator A+1 (position 34) is 34<br>distance to Mammalian Initiator A+1 (position 39) is 39
BRE upstream	59	GCTCGCC	6.18	6 out of 7	
Mammalian Initiator	56	CCAGCTC	5.82	6 out of 7	
Mammalian Initiator	34	GCATTCC	4.07	6 out of 7	
Mammalian Initiator	39	CCGGTAC	3.11	5 out of 7	
Mammalian Initiator	64	CCACCAT	2.62	5 out of 7	

>TATA_BOX_var_2-none

GGTATAAATG

No combinations of core promoter elements were detected in the input sequence.

Elements found:				
**Element**	**Start position**	**Sequence**	**PWM score**	**Consensus Match**
TATA box	3	TATAAATG	8.05	7 out of 8

>TATA_BOX_var_2-non-active

GGTATAAATAATTGGTGCCGCGAAGAAGCCTGGTTAAGGAGAACTCTCGGCGTAAGCCACCATG

No combinations of core promoter elements were detected in the input sequence.

Elements found:				
**Element**	**Start position**	**Sequence**	**PWM score**	**Consensus Match**
TATA box	3	TATAAATA	7.49	7 out of 8
Mammalian Initiator	57	CCACCAT	2.62	5 out of 7

>TATA_BOX_var_3-RND_5UTR
GGTATAAGAAGATCTGCGATCTAAGTAAGCTTGGCATTCCGGTACTGTTGGTAAACCAGCTCGCCACCATG

A possible combination, input sequence position 3 to 41:
TATAAGAAGATCTGCGATCTAAGTAAGCTTGGCATTCC

A possible combination, input sequence position 3 to 46:
TATAAGAAGATCTGCGATCTAAGTAAGCTTGGCATTCCGGTAC

Elements found:

Element	Start position	Sequence	PWM score	Consensus Match	
TATA box	3	TATAAGAA	8.05	7 out of 8	distance to Mammalian Initiator A+1 (position 34) is 34<br>distance to Mammalian Initiator A+1 (position 39) is 39
BRE upstream	59	GCTCGCC	6.18	6 out of 7	
Mammalian Initiator	56	CCAGCTC	5.82	6 out of 7	
Mammalian Initiator	34	GCATTCC	4.07	6 out of 7	
Mammalian Initiator	39	CCGGTAC	3.11	5 out of 7	
Mammalian Initiator	64	CCACCAT	2.62	5 out of 7	

>TATA_BOX_var_3-none
GGTATAAGAGCCACCATG

No combinations of core promoter elements were detected in the input sequence.

Elements found:				
**Element**	**Start position**	**Sequence**	**PWM score**	**Consensus Match**
TATA box	3	TATAAGAG	8.61	7 out of 8
Mammalian Initiator	11	CCACCAT	2.62	5 out of 7

TATA_BOX_var_3-non-active
GGTATAAGAAATTGGTGCCGCGAAGAAGCCTGGTTAAGGAGAACTCTCGGCGTAAGCCACCATG

No combinations of core promoter elements were detected in the input sequence.

Elements found:				
**Element**	**Start position**	**Sequence**	**PWM score**	**Consensus Match**
TATA box	3	TATAAGAA	8.05	7 out of 8
Mammalian Initiator	57	CCACCAT	2.62	5 out of 7

>TATA_BOX_var_4-RND_5UTR

GGTATAAGTAGATCTGCGATCTAAGTAAGCTTGGCATTCCGGTACTGTTGGTAAACCAGCTCGCCACCATG

No combinations of core promoter elements were detected in the input sequence.

Elements found:				
**Element**	**Start position**	**Sequence**	**PWM score**	**Consensus Match**
BRE upstream	59	GCTCGCC	6.18	6 out of 7
Mammalian Initiator	56	CCAGCTC	5.82	6 out of 7
Mammalian Initiator	34	GCATTCC	4.07	6 out of 7
Mammalian Initiator	39	CCGGTAC	3.11	5 out of 7
Mammalian Initiator	64	CCACCAT	2.62	5 out of 7
Mammalian Initiator	8	GTAGATC	0.89	6 out of 7

>TATA_BOX_var_4-none

GGTATAAGTGCCACCATG

No combinations of core promoter elements were detected in the input sequence.

Elements found:				
**Element**	**Start position**	**Sequence**	**PWM score**	**Consensus Match**
Mammalian Initiator	11	CCACCAT	2.62	5 out of 7
Mammalian Initiator	5	TAAGTGC	1.03	5 out of 7

>TATA_BOX_var_4-non-active

GGTATAAGTAATTGGTGCCGCGAAGAAGCCTGGTTAAGGAGAACTCTCGGCGTAAGCCACCATG

No combinations of core promoter elements were detected in the input sequence.

Elements found:				
**Element**	**Start position**	**Sequence**	**PWM score**	**Consensus Match**
Mammalian Initiator	57	CCACCAT	2.62	5 out of 7

>TATA_BOX_var_6-RND_5UTR

GGTATAGATAGATCTGCGATCTAAGTAAGCTTGGCATTCCGGTACTGTTGGTAAACCAGCTCGCCACCATG

A possible combination, input sequence position 3 to 41:

TATAGATAGATCTGCGATCTAAGTAAGCTTGGCATTCC

A possible combination, input sequence position 3 to 46:

TATAGATAGATCTGCGATCTAAGTAAGCTTGGCATTCCGGTAC

Elements found:					
**Element**	**Start position**	**Sequence**	**PWM score**	**Consensus Match**	
TATA box	3	TATAGATA	6.05	6 out of 8	distance to Mammalian Initiator A+1 (position 34) is 34<br>distance to Mammalian Initiator A+1 (position 39) is 39
BRE upstream	59	GCTCGCC	6.18	6 out of 7	
Mammalian Initiator	56	CCAGCTC	5.82	6 out of 7	
Mammalian Initiator	34	GCATTCC	4.07	6 out of 7	
Mammalian Initiator	39	CCGGTAC	3.11	5 out of 7	
Mammalian Initiator	64	CCACCAT	2.62	5 out of 7	

>TATA_BOX_var_6-none

GGTATAGATG

No combinations of core promoter elements were detected in the input sequence.

Elements found:				
**Element**	**Start position**	**Sequence**	**PWM score**	**Consensus Match**
TATA box	3	TATAGATG	6.61	6 out of 8

>TATA_BOX_var_6-non-active

GGTATAGATAATTGGTGCCGCGAAGAAGCCTGGTTAAGGAGAACTCTCGGCGTAAGCCACCATG

No combinations of core promoter elements were detected in the input sequence.

Elements found:				
**Element**	**Start position**	**Sequence**	**PWM score**	**Consensus Match**
TATA box	3	TATAGATA	6.05	6 out of 8
Mammalian Initiator	57	CCACCAT	2.62	5 out of 7

>TATA_BOX_var_9-RND_5UTR
GGTATATAAGAGATCTGCGATCTAAGTAAGCTTGGCATTCCGGTACTGTTGGTAAACCAGCTCGCCACCATG

A possible combination, input sequence position 3 to 42:
TATATAAGAGATCTGCGATCTAAGTAAGCTTGGCATTCC

A possible combination, input sequence position 5 to 42:
TATAAGAGATCTGCGATCTAAGTAAGCTTGGCATTCC

A possible combination, input sequence position 3 to 47:
TATATAAGAGATCTGCGATCTAAGTAAGCTTGGCATTCCGGTAC

A possible combination, input sequence position 5 to 47:
TATAAGAGATCTGCGATCTAAGTAAGCTTGGCATTCCGGTAC

Elements found:					
**Element**	**Start position**	**Sequence**	**PWM score**	**Consensus Match**	
TATA box	3	TATATAAG	10.86	8 out of 8	distance to Mammalian Initiator A+1 (position 35) is 35<br>distance to Mammalian Initiator A+1 (position 40) is 40
TATA box	5	TATAAGAG	8.61	7 out of 8	distance to Mammalian Initiator A+1 (position 35) is 33<br>distance to Mammalian Initiator A+1 (position 40) is 38
BRE upstream	60	GCTCGCC	6.18	6 out of 7	
Mammalian Initiator	57	CCAGCTC	5.82	6 out of 7	
Mammalian Initiator	35	GCATTCC	4.07	6 out of 7	
Mammalian Initiator	40	CCGGTAC	3.11	5 out of 7	
Mammalian Initiator	65	CCACCAT	2.62	5 out of 7	

>TATA_BOX_var_9-none

GGTATATAAGGCCACCATG

No combinations of core promoter elements were detected in the input sequence.

Elements found:				
**Element**	**Start position**	**Sequence**	**PWM score**	**Consensus Match**
TATA box	3	TATATAAG	10.86	8 out of 8
Mammalian Initiator	12	CCACCAT	2.62	5 out of 7
Mammalian Initiator	7	TAAGGCC	1.13	5 out of 7

>TATA_BOX_var_9-non-active

GGTATATAAGAATTGGTGCCGCGAAGAAGCCTGGTTAAGGAGAACTCTCGGCGTAAGCCACCATG

No combinations of core promoter elements were detected in the input sequence.

Elements found:				
**Element**	**Start position**	**Sequence**	**PWM score**	**Consensus Match**
TATA box	3	TATATAAG	10.86	8 out of 8
TATA box	5	TATAAGAA	8.05	7 out of 8
Mammalian Initiator	58	CCACCAT	2.62	5 out of 7

Extended Data Figure 1: Analysis from ElemeNT 2023

Depicted is the analysis of all minimal promoters tested in Figure 7 **(Table 7)**, done with Juven-Gershon's ElemeNT 2023 tool. Each minimal promoter is tested with RND 5'UTR, inactive 5'UTR, and without a 5'UTR.
