# Supplementary material for: Systematic engineering of synthetic minimal promoters for tunable transgene expression in mammalian cells
Source: Nucleic Acids Res. 2026 Nov 4;54(20):gkag1052. doi: 10.1093/nar/gkag1052 (PMC13732139; doi:10.1093/nar/gkag1052)
Supplement: Supplementary file 1 [file gkag1052_supplemental_files.zip › Extended Data Figure 3 Revision 3.pdf]

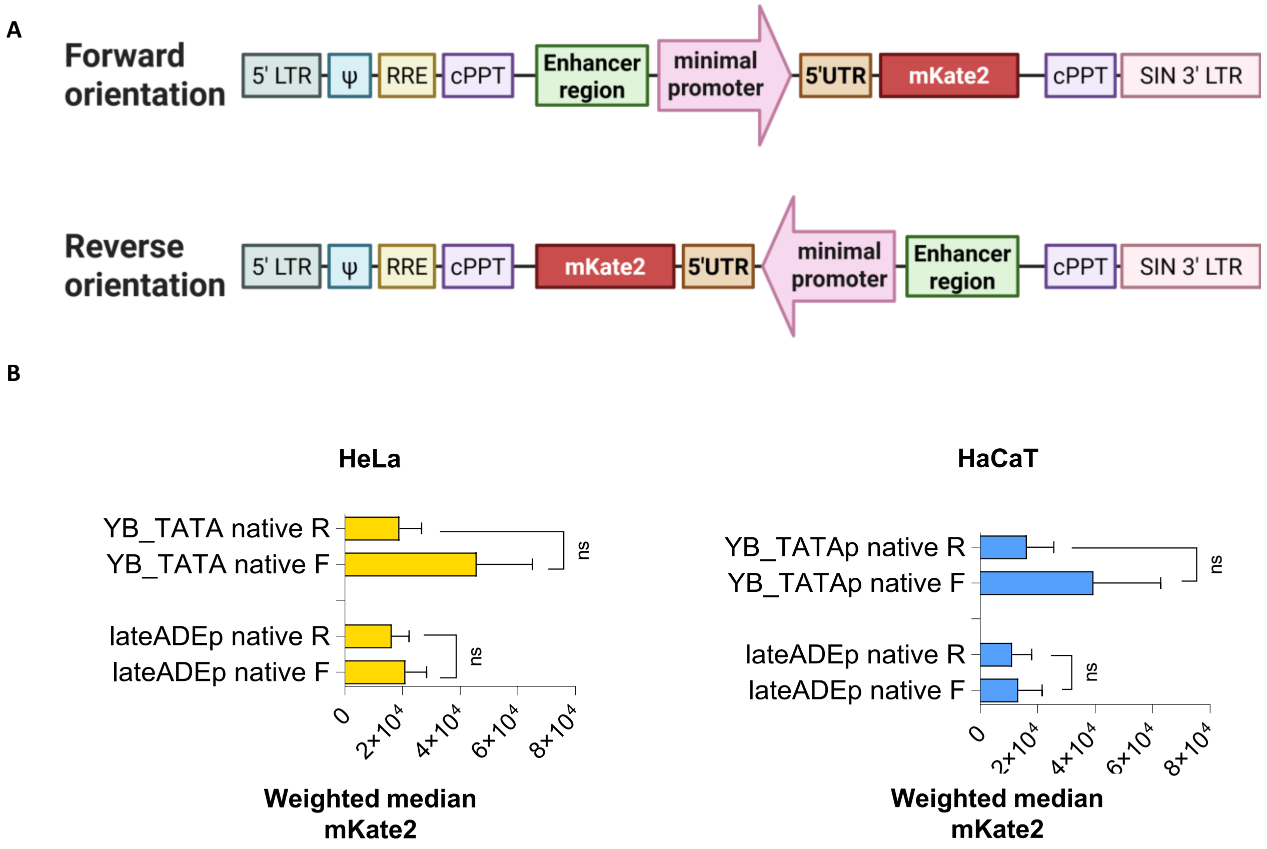

Extended Data Figure 3: Effect of insert orientation on minimal promoter activity. (A) Schematic representation of lentiviral constructs containing the enhancer-minimal promoter-5′UTR-mKate2 cassette in either the forward (top) or reverse (bottom) orientation relative to the CMVp-driven viral backbone. (B) Weighted median mKate2 expression levels of YB_TATAp and lateADEp constructs were compared between forward and reverse orientations. No significant differences were detected (ns, two-tailed t-test). Panel A was created in BioRender. Nissim, L. (2026) https://BioRender.com/sscn9yw.
