# Supplementary material for: Systematic engineering of synthetic minimal promoters for tunable transgene expression in mammalian cells
Source: Nucleic Acids Res. 2026 Nov 4;54(20):gkag1052. doi: 10.1093/nar/gkag1052 (PMC13732139; doi:10.1093/nar/gkag1052)
Supplement: Supplementary file 1 [file gkag1052_supplemental_files.zip › Supplementary Figures Revision 3 proofing 7.10.26.pdf]

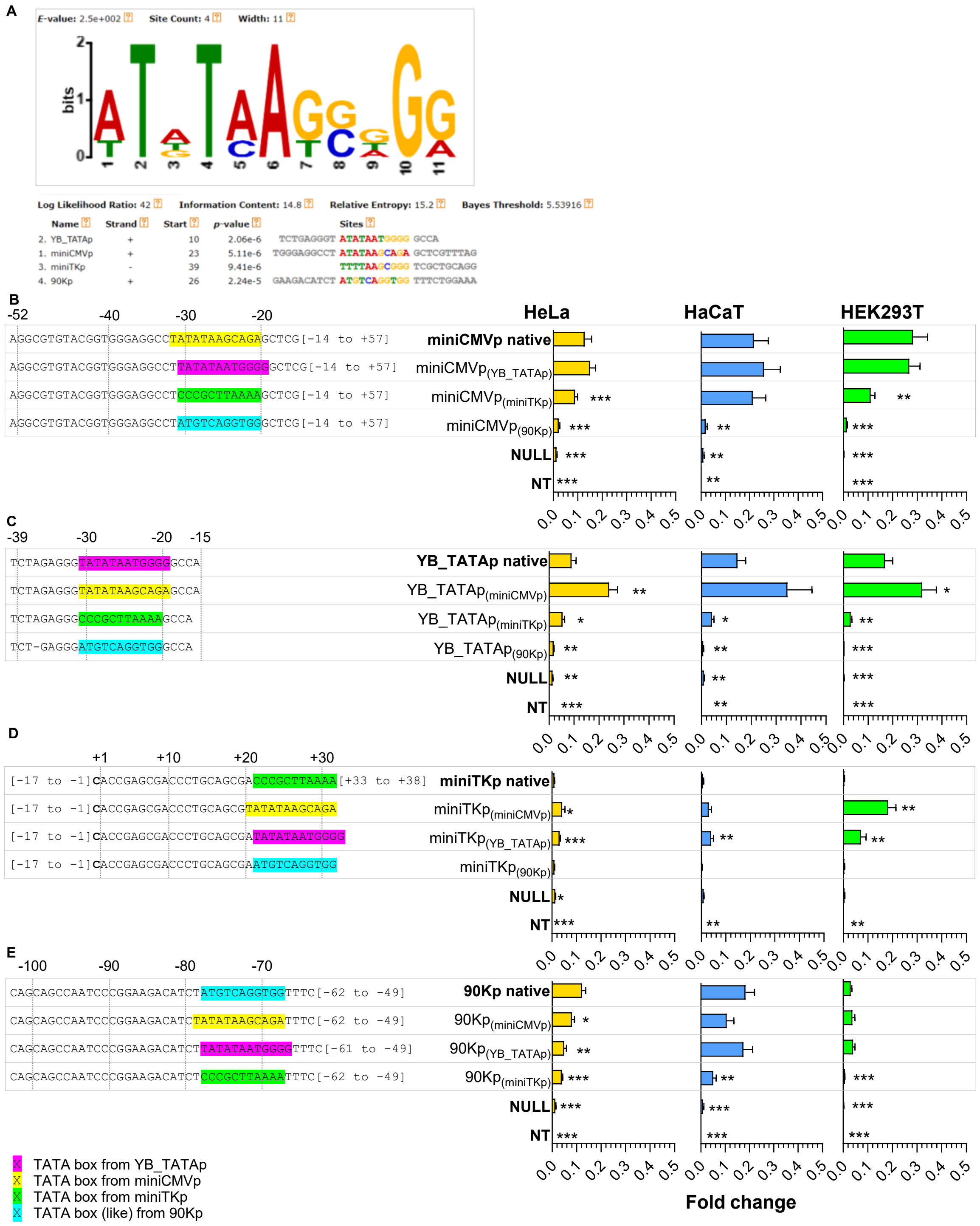

A
E-value: 2.5e+002 Site Count: 4 Width: 11
bits
Log Likelihood Ratio: 42 Information Content: 14.8 Relative Entropy: 15.2 Bayes Threshold: 5.53916
Name Strand Start p-value Sites
2. YB_TATAp + 10 2.06e-6 TCTGAGGGT ATATAATGGGG GCCA
1. miniCMVp + 23 5.11e-6 TGGGAGGCCT ATATAAGCAGA GCTCGTTTAG
3. miniTKp - 39 9.41e-6 TTTTAAGCGGG TCGCTGCAGG
4. 90Kp + 26 2.24e-5 GAAGACATCT ATGTCAGGTGG TTTCTGGAAA
B
HeLa
HaCaT
HEK293T
AGGCGTGTACGGTGGGAGGCCTATATAAGCAGAGCTCG[-14 to +57]
AGGCGTGTACGGTGGGAGGCCTTATATAATGGGGGCTCG[-14 to +57]
AGGCGTGTACGGTGGGAGGCCTCCCGCTTAAAAGCTCG[-14 to +57]
AGGCGTGTACGGTGGGAGGCCTATGTCAGGTGGGCTCG[-14 to +57]
miniCMVp native
miniCMVp(YB_TATAp)
miniCMVp(miniTKp)
miniCMVp(90Kp)
NULL
NT
C
TCTAGAGGGTATATAATGGGGGCCA
TCTAGAGGGTATATAAGCAGAGCCA
TCTAGAGGGCCCGCTTAAAAGCCA
TCT-GAGGGATGTCAGGTGGGCCA
YB_TATAp native
YB_TATAp(miniCMVp)
YB_TATAp(miniTKp)
YB_TATAp(90Kp)
NULL
NT
D
[-17 to -1]CACCGAGCGACCCTGCAGCGACCCGCTTAAAA[+33 to +38]
[-17 to -1]CACCGAGCGACCCTGCAGCGTATATAAGCAGA
[-17 to -1]CACCGAGCGACCCTGCAGCGATATATAATGGGG
[-17 to -1]CACCGAGCGACCCTGCAGCGAATGTCAGGTGG
miniTKp native
miniTKp(miniCMVp)
miniTKp(YB_TATAp)
miniTKp(90Kp)
NULL
NT
E
CAGCAGCCAATCCCGGAAGACATCTATGTCAGGTGGTTTC[-62 to -49]
CAGCAGCCAATCCCGGAAGACATCTATATAAGCAGATTTC[-62 to -49]
CAGCAGCCAATCCCGGAAGACATCTTATATAATGGGGTTTC[-61 to -49]
CAGCAGCCAATCCCGGAAGACATCTCCCGCTTAAAATTTC[-62 to -49]
90Kp native
90Kp(miniCMVp)
90Kp(YB_TATAp)
90Kp(miniTKp)
NULL
NT
Fold change
TATA box from YB_TATAp
TATA box from miniCMVp
TATA box from miniTKp
TATA box (like) from 90Kp

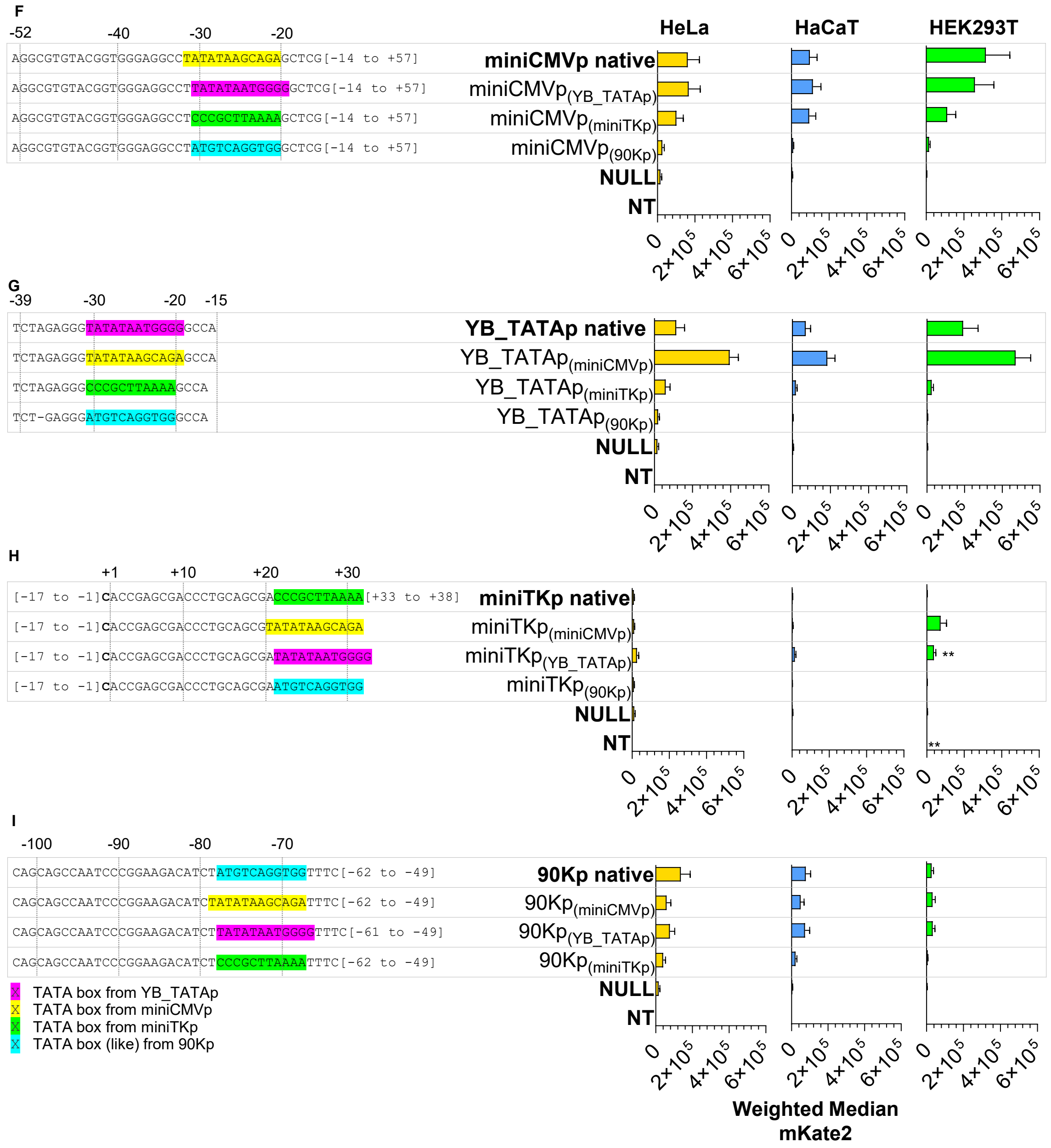

Figure S1 (Related to Figure 1): TATA box Swapping between Minimal Promoters Reveals Context-Dependent Effects on Promoter Activity

(A) Analysis of four minimal promoters (miniCMVp, YB_TATAp, miniTKp, 90Kp) with the MEME motif identifier (75) to find the sequences most similar between all promoters. **(B-E)** The graph shows the weighted median mKate2 expressed under each unique minimal promoter construct, relative to a control hUbCp-mKate2 weighted median. **(F-I)** The graph shows the absolute weighted median of mKate2 expressed under each unique minimal promoter construct. All minimal promoter variants are annotated relative to the native minimal promoter's transcription start site (TSS; +1).

NULL denotes the lack of a minimal promoter within the synthetic enhancer-reporter construct; NT denotes non-treated, naïve cells; square brackets indicate additional downstream nucleotides in the minimal promoter, with the position of non-displayed nucleotides shown in brackets (full sequences in Table 1). Data are presented as the mean±SEM; n≥3 biological replicates; ∗p< 0.05, **p<0.01, ***p<0.001 by multiple unpaired t-test, FDR 5% against native promoter. Related to Figure 1.

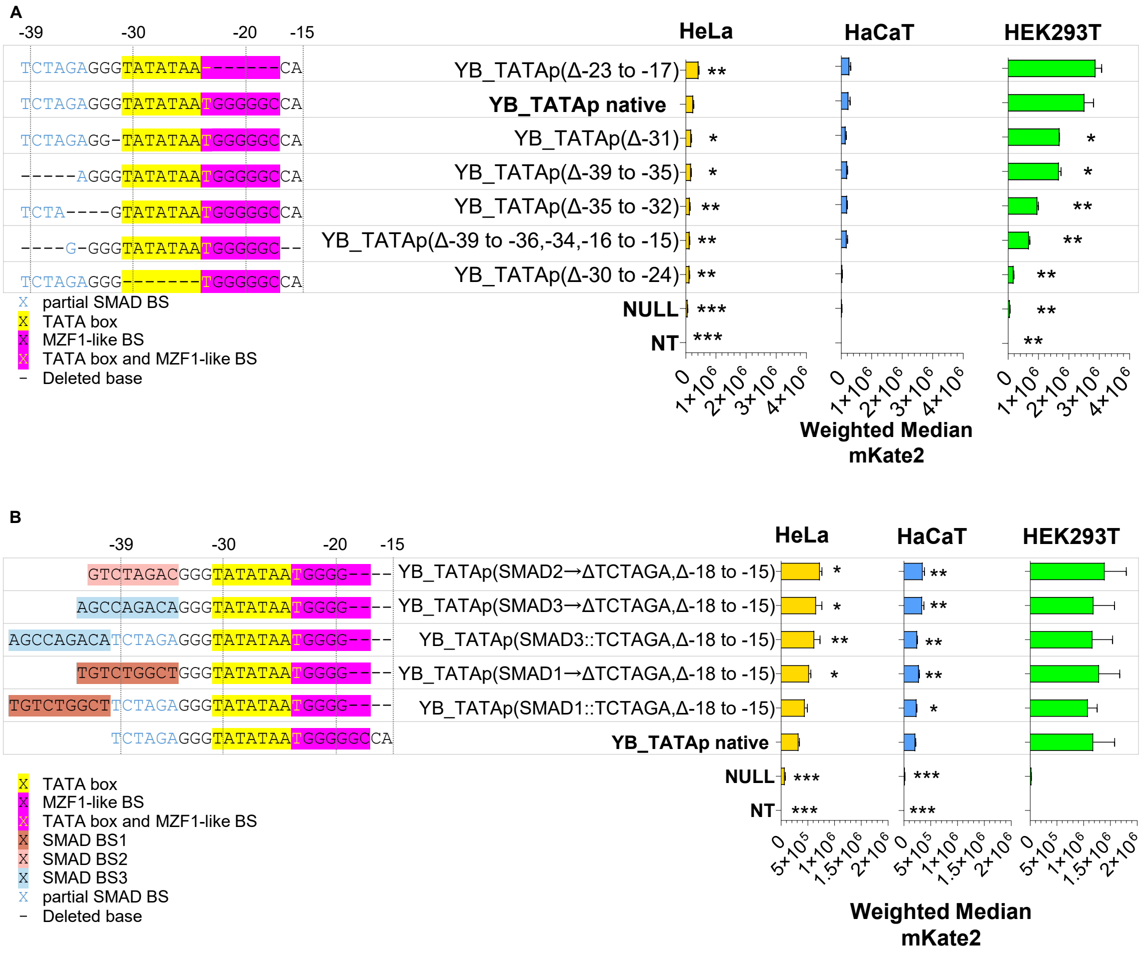

Figure S2 (Related to Figure 2): Deletion Analysis and Remodeling of the YB_TATA Promoter Reveals Sequence Features that Fine-Tune Expression via Repression or Enhancement (Absolute mKate2 Fluorescence)

(A) Modulatory mutational analysis of YB_TATAp elements reveals sequence features that are varying contributions to expression in HeLa, HaCaT and HEK293T cells**. (B)** The partial SMAD binding site (BS) motif (TCTAGA) was either replaced or placed downstream of various known full SMAD binding sites. For both panels, graphs show weighted median mKate2 fluorescence presented in descending order of magnitude per HeLa cells.

- denotes deleted nucleotides; NULL denotes the lack of a minimal promoter within the synthetic enhancer-reporter construct; NT denotes non-treated, naïve cells. All minimal promoter variants are annotated relative to the native minimal promoter's transcription start site (TSS; +1). Data are presented as the mean±SEM; n≥3 biological replicates; ∗$p<0.05$, ∗∗$p<0.01$, ∗∗∗$p<0.001$ by multiple unpaired t-test, FDR 5% against YB_TATAp native. Related to Figure 2.

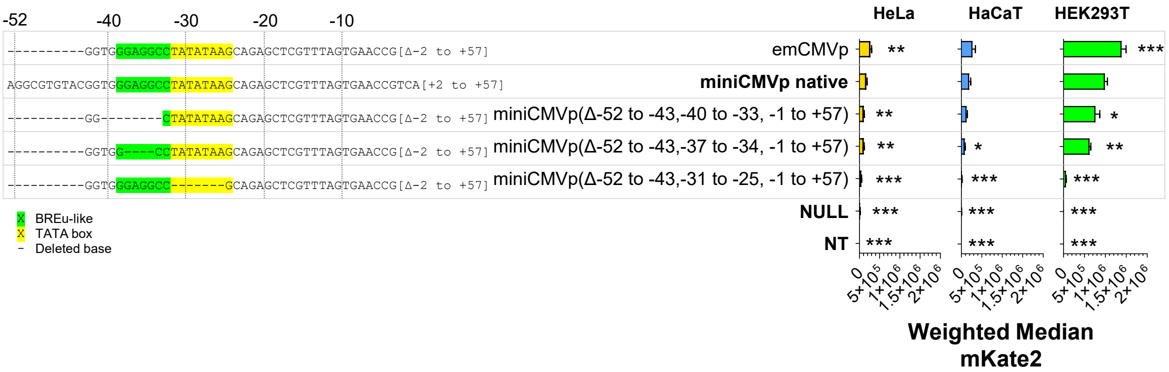

Figure S3 (Related to Figure 3): Deletions in the miniCMVp Show Sequence Redundancy and Maps Promoter Motif Regions Associated with Increased or Decreased Output Across Multiple Human Cell types (Absolute mKate2 Fluorescence)

Systematic deletion of identified elements within the miniCMVp reveals varied effects on expression, with enhanced activity following deletion of nucleotides upstream of the first identified element, in HeLa, HaCaT and HEK293T cells. The graph shows the weighted median mKate2, presented in descending order of magnitude per HeLa cells.

- denotes deleted nucleotides; NULL denotes the lack of a minimal promoter within the synthetic enhancer-reporter construct; NT denotes non-treated, naïve cells. All minimal promoter variants are annotated relative to the native minimal promoter's transcription start site (TSS; +1). Data are presented as the mean±SEM; n≥6 biological replicates; $*p< 0.05$, $**p<0.01$, $***p<0.001$ by multiple unpaired t-test, FDR 5% against miniCMVp native. Related to Figure 3.

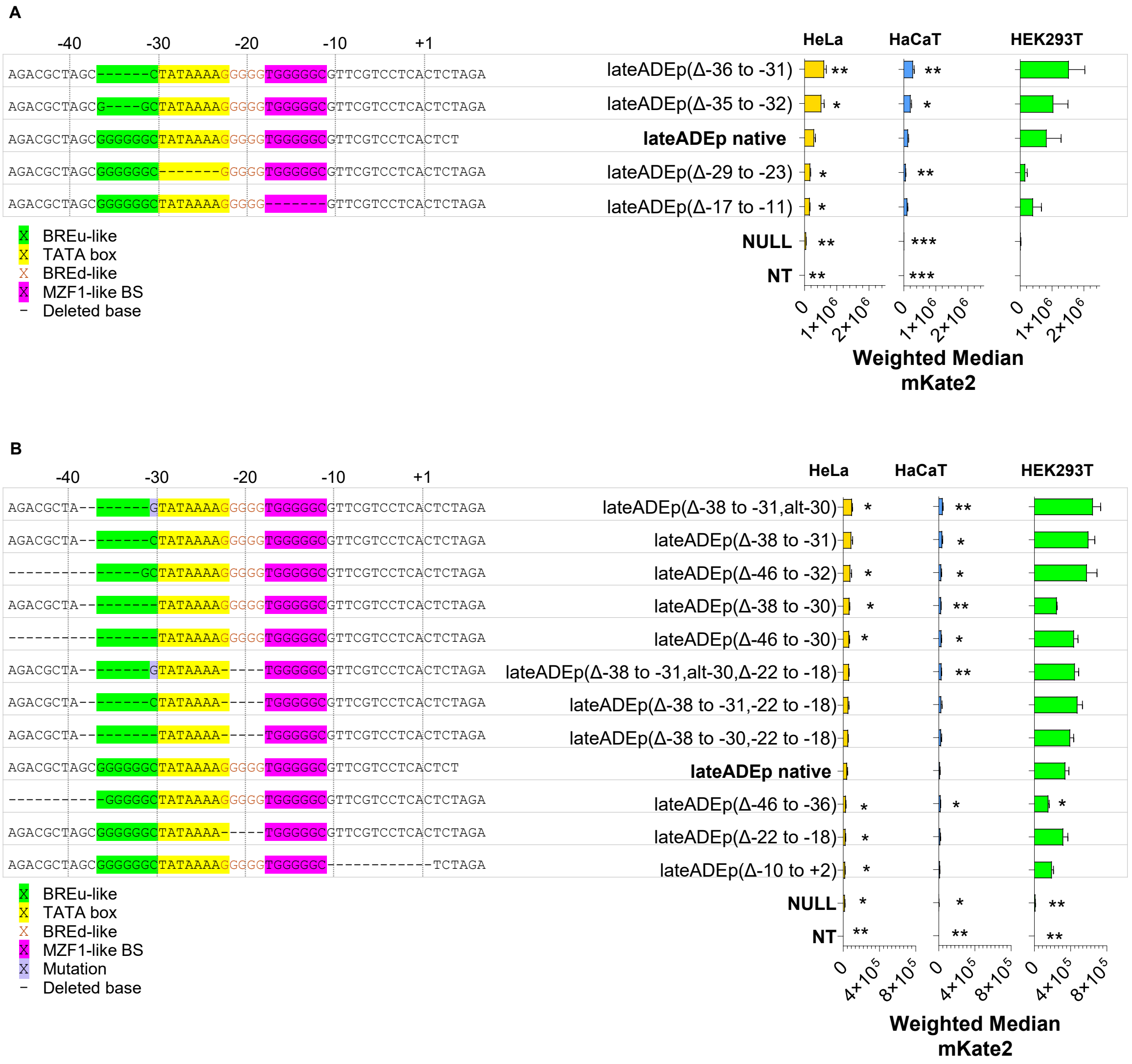

Figure S4 (Related to Figure 4): Targeted Deletional Analysis of the Adenovirus Major Late Promoter (lateADEp) Reveals Distinct Sequence Features that Enhance or Repress Expression (Absolute mKate2 Fluorescence)

(A) Weighted median mKate2 fluorescence following systematic element deletions. **(B)** Combinatorial deletions and mutations were applied additionally to the BREu-like motif region deletions in HeLa, HaCaT and HEK293T cells. For both panels, graphs show weighted median mKate2 fluorescence presented in descending order of magnitude per HeLa cells.

- denotes deleted nucleotides; NULL denotes the lack of a minimal promoter within the synthetic enhancer-reporter construct; NT denotes non-treated, naïve cells. All minimal promoter variants are annotated relative to the native minimal promoter's transcription start site (TSS; +1). Data are presented as the mean±SEM; n≥3 biological replicates; ∗p<0.05,

**p<0.01, ***p<0.001 by multiple unpaired t-test, FDR 5% against lateADEp native. Related to Figure 4.

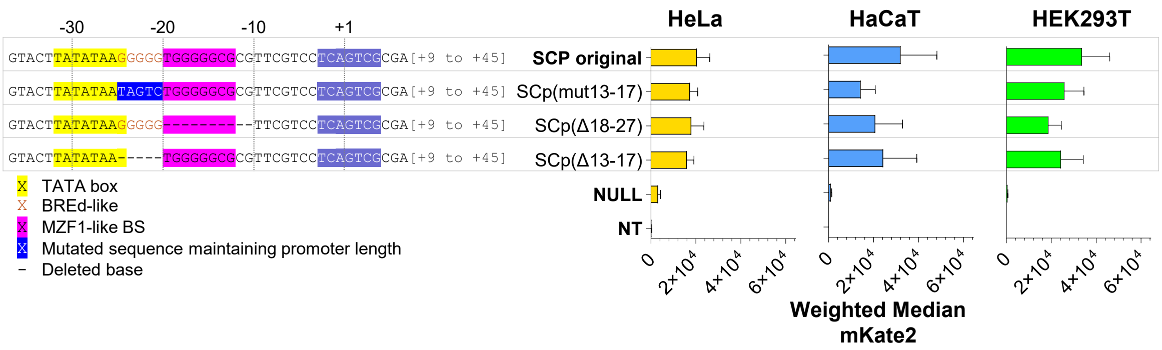

Figure S5 (Related to Figure 5): Modulatory Mutational and Deletion Analysis of Elements within the Synthetic Super Minimal promoter (SCP) Results in Minimal Changes (Absolute mKate2 Fluorescence)

The graph shows the weighted median mKate2 fluorescence in HeLa, HaCaT and HEK293T cells, presented in descending order of magnitude per HeLa cells.

- denotes deleted nucleotides; NULL denotes the lack of a minimal promoter within the synthetic enhancer-reporter construct; NT denotes non-treated, naïve cells. All minimal promoter variants are annotated relative to the native minimal promoter's transcription start site (TSS; +1). Data are presented as the mean±SEM; n=3 biological replicates; ∗p<0.05, **p<0.01, ***p<0.001 by multiple unpaired t-test, FDR 5% against SCP original. Related to Figure 5.

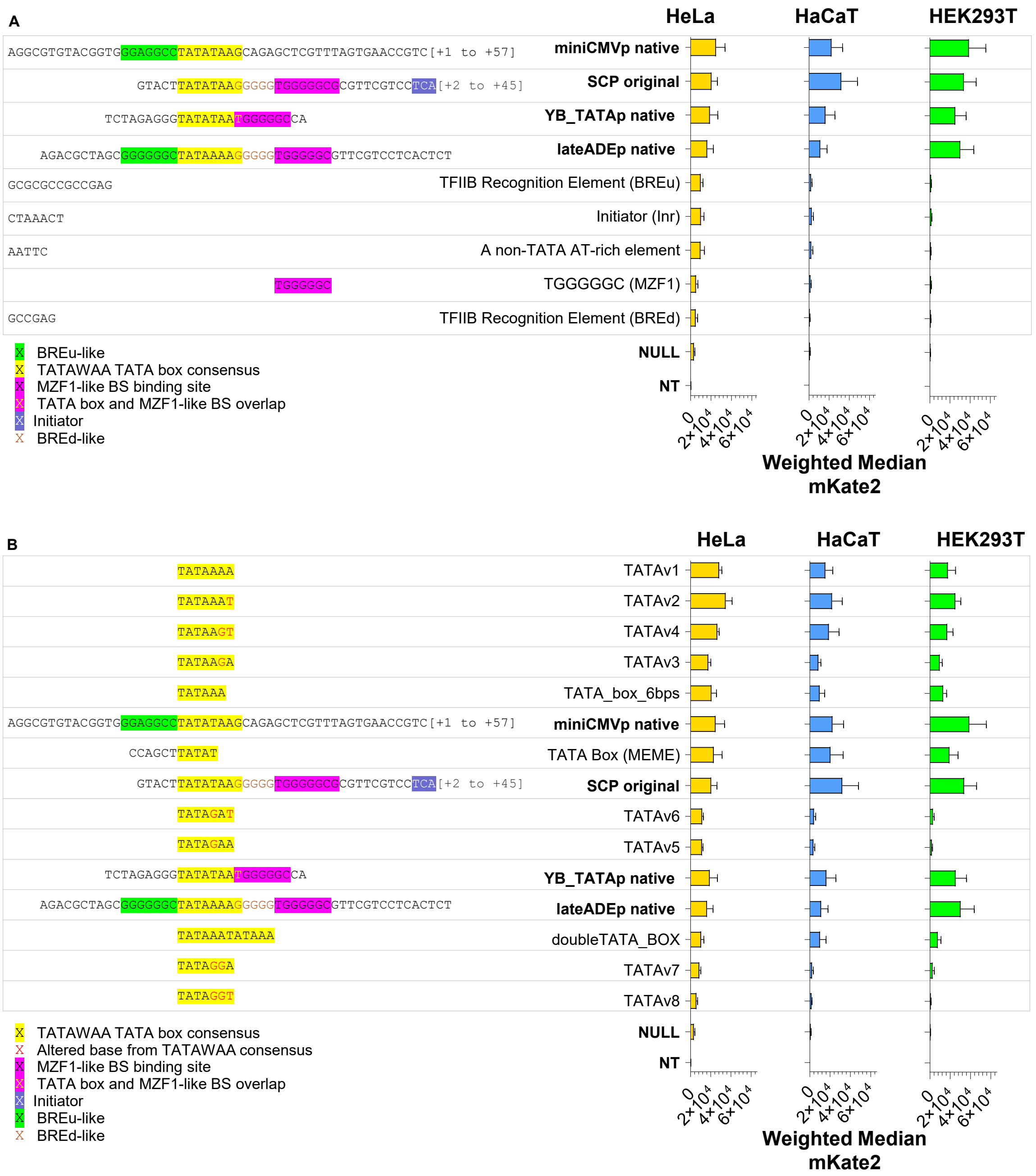

Figure S6 (Related to Figure 6): Isolating Minimal promoter Elements and Systematic Mutagenesis of TATA boxes Reveals High-Activity TATA box Variants and Gradient Expression Profiles (Absolute mKate2 Fluorescence)

(A) Constructs containing a single element, such as an Initiator (Inr), DCE, or a TFIIB recognition element, inserted in the minimal promoter position. **(B)** Constructs containing only a TATA box inserted in the minimal promoter position. Several TATA box variants were included (strategy for TATA box mutagenesis in Table 3). For both panels, graphs show weighted median mKate2 expression in HeLa, HaCaT and HEK293T cells, presented in descending order of magnitude per HeLa cells. NULL denotes the lack of a minimal promoter within the synthetic enhancer-

reporter construct; NT denotes non-treated, naïve cells. All minimal promoter variants are annotated relative to the native minimal promoter's transcription start site (TSS; +1). Data are presented as the mean±SEM; n=3 biological replicates; ∗$p<0.05$, **$p<0.01$, ***$p<0.001$ by multiple unpaired t-test, FDR 5% against miniCMVp native. Related to Figure 6.

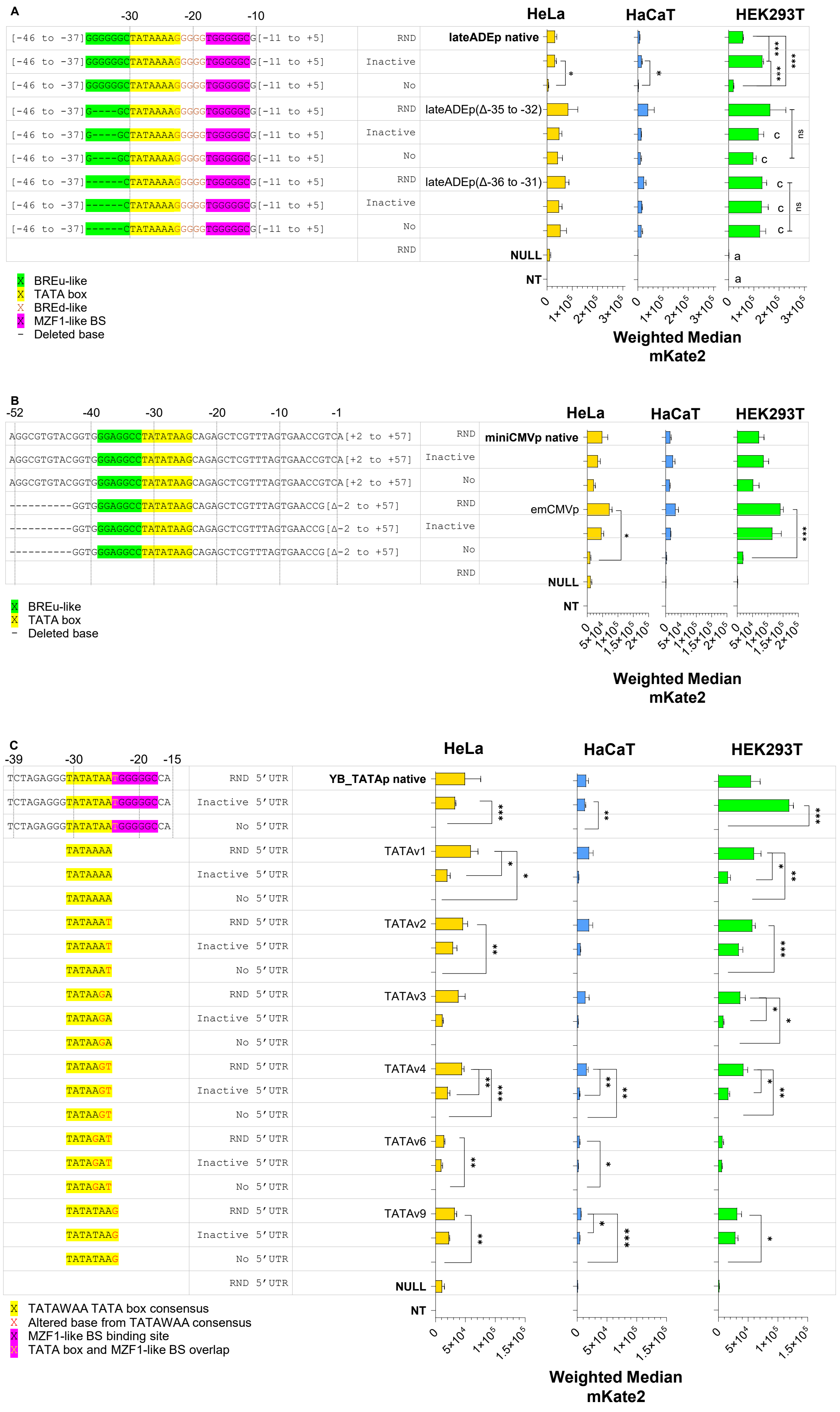
A
-30 -20 -10
HeLa
HaCaT
HEK293T
[-46 to -37]GGGGGGCTATAAAAGGGGGTGGGGGCG[-11 to +5]
[-46 to -37]G----GCTATAAAAGGGGGTGGGGGCG[-11 to +5]
[-46 to -37]------CTATAAAAGGGGGTGGGGGCG[-11 to +5]
RND
Inactive
No
lateADEp native
lateADEp(Δ-35 to -32)
lateADEp(Δ-36 to -31)
NULL
NT
BREu-like
TATA box
BREd-like
MZF1-like BS
Deleted base
Weighted Median mKate2
B
-52 -40 -30 -20 -10 -1
AGGCGTGTACGGTGGGAGGCCTATATAAGCAGAGCTCGTTTAGTGAACCGTCA[+2 to +57]
----------GGTGGGAGGCCTATATAAGCAGAGCTCGTTTAGTGAACCG[Δ-2 to +57]
miniCMVp native
emCMVp
BREu-like
TATA box
Deleted base
C
-39 -30 -20 -15
TCTAGAGGGTATATAATGGGGGCCA
TATAAAA
TATAAAT
TATAAGA
TATAAGT
TATAGAT
TATATAAG
RND 5′UTR
Inactive 5′UTR
No 5′UTR
YB_TATAp native
TATAv1
TATAv2
TATAv3
TATAv4
TATAv6
TATAv9
TATAWAA TATA box consensus
Altered base from TATAWAA consensus
MZF1-like BS binding site
TATA box and MZF1-like BS overlap

Figure S7 (Related to Figure 7): 5' UTR Sequence Modulates Transcription in Short Versus Long Minimal promoter Constructs (Absolute mKate2 Fluorescence)

(A) Constructs containing previously designed lateADEp variants with different 5'UTR sequences. **(B)** Constructs a previously designed miniCMVp variant with different 5'UTR sequences in HeLa, HaCaT and HEK293T cells. For all panels, graphs show weighted median mKate2 expression presented in groups by promoter variant. **(C)** Constructs containing a TATA box inserted in the minimal promoter position evaluated with different 5'UTR sequences. Several TATA box variants were included (strategy for TATA box mutagenesis in Table 3).

NULL denotes the lack of a minimal promoter within the synthetic enhancer-reporter construct; NT denotes non-treated, naïve cells. All minimal promoter variants are annotated relative to the native minimal promoter's transcription start site (TSS; +1). Data are presented as the mean±SEM; n≥3 biological replicates; ∗p<0.05, **p<0.01, ***p<0.001; [c] p< 0.05 [b] p<0.01, [a] p<0.001, all analyzed by multiple unpaired t-test, FDR 5% against native (lateADEp, miniCMVp, YB_TATAp). Related to Figure 7.

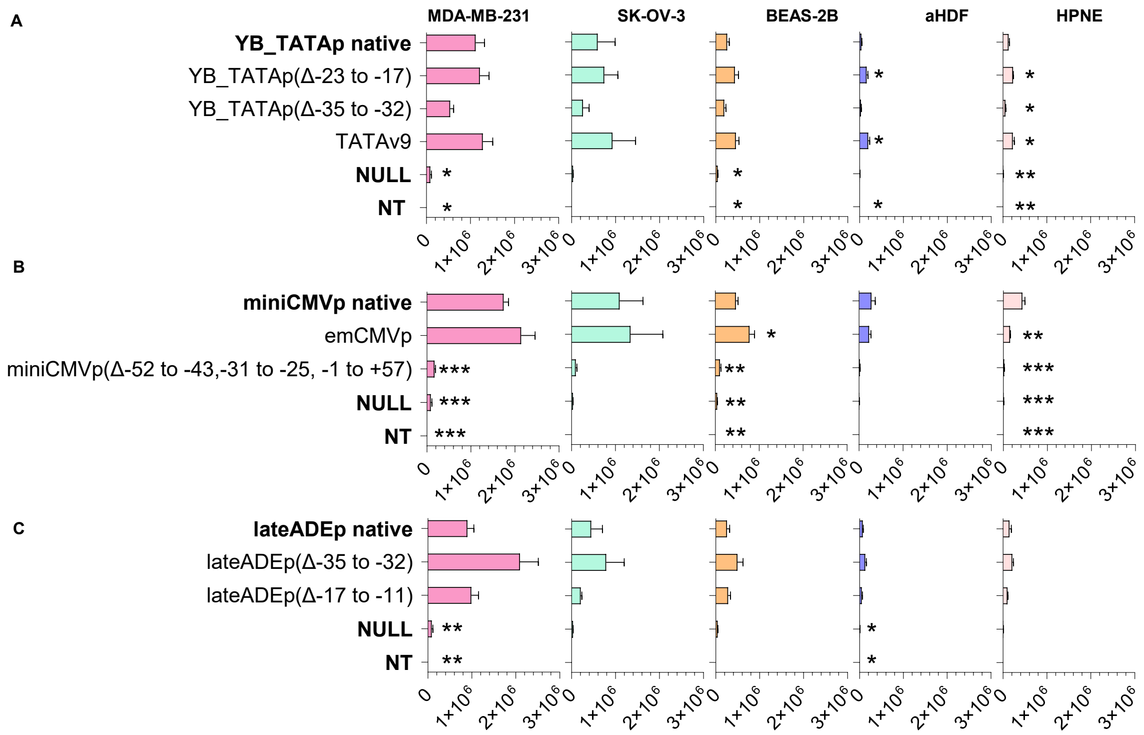

Figure S8 (Related to Figure 8): Assessment of Different Minimal Promoters Across a Diverse Panel of Human Cell Types (Absolute mKate2 Fluorescence)

Representative minimal promoter variants were selected from the lateADEp, YB_TATAp, and miniCMVp groups to evaluate their functionality in five additional human cell types representing malignant and non-malignant cells from distinct tissues. For each architecture, two to three constructs were characterized across five additional human cell lines (MDA-MB-231, SK-OV-3, BEAS-2B, aHDF, and HPNE). (A) YB_TATAp variants, (B) miniCMVp variants, and (C) lateADEp variants. Graphs show absolute weighted median mKate2 fluorescence.

Full minimal promoter variation sequences are detailed in Table 4. - denotes deleted nucleotides; NULL denotes the lack of a minimal promoter within the synthetic enhancer-reporter construct; NT denotes non-treated, naïve cells. Data are presented as the mean±SEM; n=3 biological replicates; ∗p< 0.05, **p<0.01, ***p<0.001 by multiple unpaired t-test, FDR 5% against each native. Related to Figure 8.

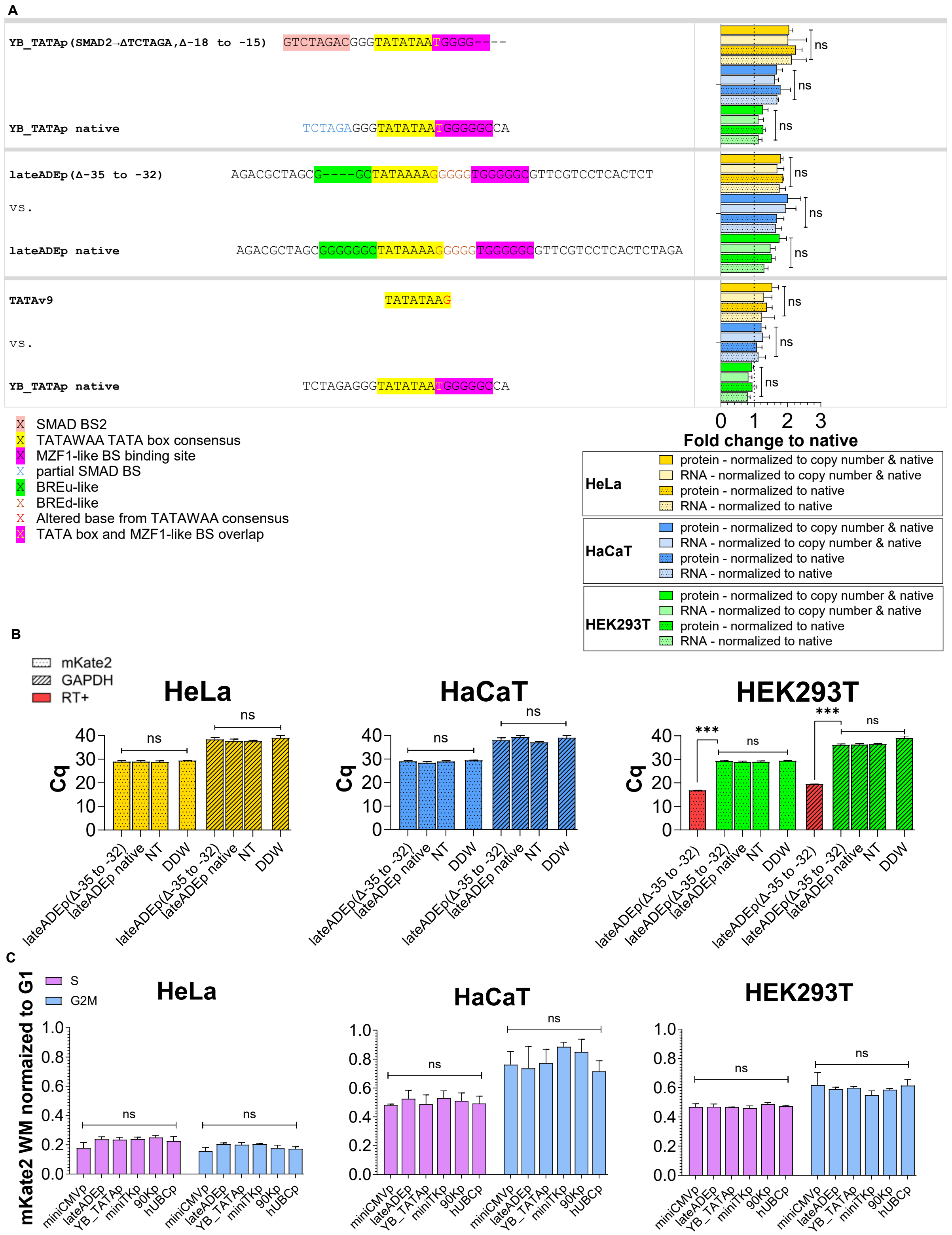

Figure S9: RNA-Output Validation, Copy-Number Normalization, and Cell-Cycle Dependency of Minimal Promoter Activity

(A) Differences in synthetic minimal promoter activity reflect transcriptional efficiency rather than viral transduction efficiency. RNA and protein output for three representative minimal

promoter variants in HeLa, HaCaT, and HEK293T cells. Bars show fold change relative to the corresponding native promoter (native = 1) in HeLa, HaCaT, and HEK293T cells, reporting both protein and RNA levels under two normalization schemes: normalization to vector copy number and the corresponding native promoter, or normalization to the corresponding native promoter. Data are presented as the mean ± SEM of n = 3 independent biological replicates of independent lentiviral transductions, with each biological replicate comprising n=3 technical qPCR replicates of gDNA and RNA. Data are presented as the mean±SEM; n=3 biological replicates; ns = non-significant by One-way ANOVA followed by Tukey's multiple-comparisons test. **(B)** qPCR Cq values for mKate2 (dotted bars) and GAPDH (diagonal bars) in cells expressing the lateADEp(Δ-35 to -32) or lateADEp native variants. No-reverse-transcriptase (RT-) reactions were run for HeLa, HaCaT, and HEK293T samples, and for non-transduced cells (NT), using the standard reaction without addition of reverse transcriptase. Double-distilled water (DDW) served as a no-template negative control. As a positive control, one HEK293T lateADEp(Δ-35 to -32) RNA sample was processed with reverse transcriptase (RT+, red) and quantified alongside the corresponding RT- samples. Reactions that did not reach the detection threshold within the 40-cycle limit were assigned a Cq of 40 for display and analysis. Data are presented as the mean ± SEM of n = 3 independent biological replicates of independent lentiviral transductions, with each biological replicate comprising n=3 technical qPCR replicates of RNA; ns = non-significant, ∗p<0.05, **p<0.01, ***p<0.001 by one-way ANOVA followed by Tukey's multiple-comparisons test. **(C)** Evaluation of synthetic minimal promoter activity across cell-cycle phases. Graphs show the mKate2 weighted median in S and G2/M phases, normalized to the corresponding G1-phase weighted median. Data are presented as the mean±SEM; n = 3 biological replicates; ns = non-significant. One-way ANOVA followed by Tukey's multiple-comparisons test compared mKate2 expression levels among different minimal promoters within each cell-cycle stage.
